# Comprehensive Multi-omics Analysis of Regulatory Variants for Body Weight in Cattle

**DOI:** 10.1093/gpbjnl/qzaf067

**Published:** 2025-08-18

**Authors:** Qunhao Niu, Jiayuan Wu, Tianyi Wu, Tianliu Zhang, Tianzhen Wang, Xu Zheng, Zhida Zhao, Ling Xu, Zezhao Wang, Bo Zhu, Lupei Zhang, Huijiang Gao, George E Liu, Junya Li, Lingyang Xu

**Affiliations:** State Key Laboratory of Animal Biotech Breeding, Institute of Animal Science, Chinese Academy of Agricultural Sciences, Beijing 100193, China; State Key Laboratory of Animal Biotech Breeding, Institute of Animal Science, Chinese Academy of Agricultural Sciences, Beijing 100193, China; State Key Laboratory of Animal Biotech Breeding, Institute of Animal Science, Chinese Academy of Agricultural Sciences, Beijing 100193, China; State Key Laboratory of Animal Biotech Breeding, Institute of Animal Science, Chinese Academy of Agricultural Sciences, Beijing 100193, China; College of Animal Science and Technology, Henan Agricultural University, Zhengzhou 450046, China; State Key Laboratory of Animal Biotech Breeding, Institute of Animal Science, Chinese Academy of Agricultural Sciences, Beijing 100193, China; State Key Laboratory of Animal Biotech Breeding, Institute of Animal Science, Chinese Academy of Agricultural Sciences, Beijing 100193, China; State Key Laboratory of Animal Biotech Breeding, Institute of Animal Science, Chinese Academy of Agricultural Sciences, Beijing 100193, China; School of Biomedical Sciences, Li Ka Shing Faculty of Medicine, The University of Hong Kong, Hong Kong Special Administrative Region 999077, China; State Key Laboratory of Animal Biotech Breeding, Institute of Animal Science, Chinese Academy of Agricultural Sciences, Beijing 100193, China; State Key Laboratory of Animal Biotech Breeding, Institute of Animal Science, Chinese Academy of Agricultural Sciences, Beijing 100193, China; State Key Laboratory of Animal Biotech Breeding, Institute of Animal Science, Chinese Academy of Agricultural Sciences, Beijing 100193, China; State Key Laboratory of Animal Biotech Breeding, Institute of Animal Science, Chinese Academy of Agricultural Sciences, Beijing 100193, China; Animal Genomics and Improvement Laboratory, United States Department of Agriculture-Agricultural Research Services, Beltsville, MD 20705, USA; State Key Laboratory of Animal Biotech Breeding, Institute of Animal Science, Chinese Academy of Agricultural Sciences, Beijing 100193, China; State Key Laboratory of Animal Biotech Breeding, Institute of Animal Science, Chinese Academy of Agricultural Sciences, Beijing 100193, China

**Keywords:** Body weight, Multi-omics, Genetic regulation, Functional variant, Cattle

## Abstract

Body weight is a polygenic trait with intricate inheritance patterns. Functional genomics enriched with multi-layer annotations offers essential resources for exploring the genetic architecture of complex traits. In this study, we conducted an extensive characterization of regulatory variants associated with body weight-related traits in cattle using multi-omics analysis. First, we identified seven candidate genes by integrating selective sweep analysis and multiple genome-wide association study (GWAS) strategies using imputed whole-genome sequencing data from a population of 1577 individuals. Subsequently, we uncovered 3340 eGenes (genes whose expression levels are associated with genetic variants) across 227 muscle samples. Transcriptome-wide association studies (TWASs) further revealed a total of 532 distinct candidate genes associated with body weight-related traits. Colocalization analyses unveiled 44 genes shared between expression quantitative trait loci (eQTLs) and GWAS signals. Moreover, a comprehensive analysis by integrating GWAS, selective sweep, eQTL, TWAS, epigenomic profiling, and molecular validation highlighted a positively selected genomic region on *Bos taurus* autosome 6 (BTA6). This locus harbors pleiotropic genes (*LAP3*, *MED28*, and *NCAPG*) and a prioritized functional variant involved in the complex regulation of body weight. Additionally, convergent evolution analysis and phenome-wide association studies underscored the conservation of this locus across species. Our study provides a comprehensive understanding of the genetic regulation of body weight through multi-omics analysis in cattle. Our findings contribute to unraveling the genetic mechanisms governing weight-related traits and shed valuable light on the genetic improvement of farm animals.

## Graphical abstract

**Figure:**
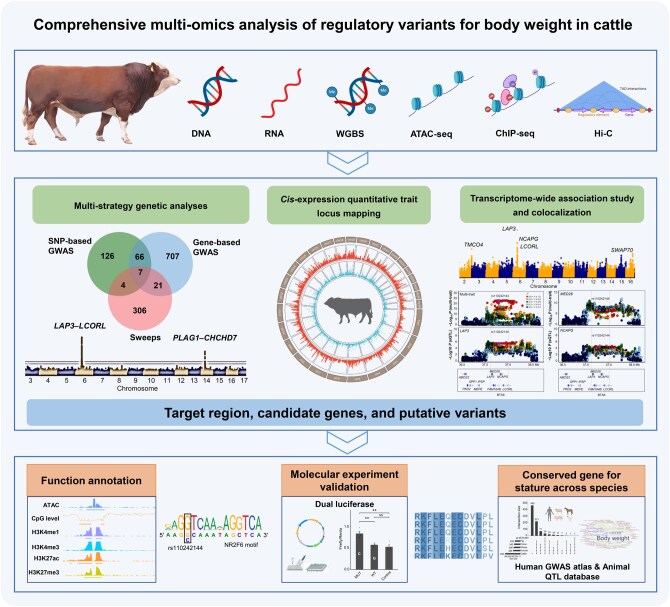

## Introduction

Body weight, with its polygenic nature, is one of the crucial indicators of production performance in domestic species [1]. The development of genome-wide association studies (GWASs) has greatly expanded our knowledge of the genetic factors influencing body weight and size. Extensive genome-wide scans have unveiled selective genomic regions and candidate variants affecting body weight in various species [2–8], underscoring the conservation of their effects on body weight and size across mammals [1,9]. A recent study has identified hundreds of putative loci that explain up to 13.8% of the phenotypic variance in cattle stature and demonstrated potential consensus associations across species, including dogs and humans [10,11]. Large-scale GWASs in various cattle breeds have identified hundreds of variants in genes such as *MSTN* and *LCORL*, which are associated with weight-related traits like growth, morphology, and carcass characteristics [12–14]. These identified single nucleotide polymorphisms (SNPs) are enriched in genes, pathways, and regulatory elements in specific tissue types, likely reflecting multiple annotations of the key biological mechanisms underlying variant functions. This information helps us comprehend the role of genetic variation in biological architecture that shapes complex phenotypes [15–19].

The advent of multi-omics approaches has revolutionized our understanding of the functional genetic variants underlying complex traits [17,20–22]. International initiatives including the Genotype-Tissue Expression (GTEx) project [23,24], the Encyclopedia of DNA Elements (ENCODE) project [25], the Functional ANnotation of Animal Genomes (FAANG) project [26], and CattleGTEx [27] have been instrumental in elucidating epigenomic and transcriptomic landscapes across diverse tissues and cell types in both humans and farm animals. Numerous studies have emphasized that GWAS hits associated with complex traits are prominently enriched in regulatory regions, such as promoters and enhancers, indicating their critical role in explaining the missing heritability [28–30]. This presents an opportunity to elucidate the genetic basis of important traits through multi-omics profiling and fine-mapping of causal variants [31,32]. In recent years, the integration of multi-omics data has led to the identification of numerous variants associated with human stature, underscoring the influence of functional regulatory elements on the regulation of complex traits [15,33,34].

In farm animals, numerous candidate variants within essential genes associated with body weight, size, and growth have been identified through genome-wide scanning methods, including GWAS and selective sweep analyses [5,10,35]. A previous study has reported that *cis*-regulatory elements contributed to the selection and adaptive evolution of modern breeds [36], facilitating the identification of key loci with functional regulatory roles in economically important traits. For instance, multi-strategy approaches have identified the *NCAPG* region as a candidate locus for average daily gain and carcass weight in cattle [37,38]. The *NCAPG–LCORL* loci have been further implicated by selective sweeps across five cattle breeds for stature using meta-analysis [10], and have been previously identified as common genes regulating body size in mammals [39]. Additionally, expression analyses have revealed that quantitative trait loci (QTLs) affecting tenderness and growth are most likely *cis*-expression QTLs (*cis*-eQTLs) for *LCORL* [40]. Moreover, another study has suggested that an expression QTL (eQTL) for *PLAG1*, underlying major pleiotropic effects, also shows associations with body weight and growth [41]. Many studies advocate for the use of multi-omics strategies to analyze the genetic basis of complex traits and elucidate the mechanisms of trait formation in pigs [42], sheep [43], and cattle [44]. However, most of the datasets are derived from tissues comprising heterogeneous cell populations, which hinders the resolution of functional information and limits our understanding of the fundamental processes underlying phenotypes [45]. Thus, a comprehensive exploration of the potential regulatory roles of candidate variants underlying body weight and size by integrating gene expression and functional elements remains necessary.

In our study, we employed a multifaceted approach, combining GWAS and selective sweep analyses, to investigate body weight-related traits using sequencing variants in cattle. Through a series of analyses, including eQTL mapping, transcriptome-wide association studies (TWASs), colocalization, and summary-data-based Mendelian randomization (SMR), we identified and prioritized candidate genes along with their associated variants. Moreover, we carried out a comprehensive analysis of variant–gene pairs to explore their functional regulatory patterns. Our findings pinpointed a genomic region, covering genes like *LAP3*, *MED28*, and *NCAPG*, associated with body weight, supported by strong evidence from multiple strategies and multi-omics approaches. Our study enhances the understanding of regulatory variations influencing economically important traits and sheds light on the selection and genetic mechanisms governing complex traits in farm animals.

## Results

### Data summary

In this study, we integrated multi-omics data including genomic, transcriptomic, and epigenomic data to elucidate the genetic regulation for body weight-related traits. The workflow of the current study is provided in Figure S1. First, we imputed Illumina BovineHD SNP arrays to the sequence level, utilizing Run8 of the 1000 Bull Genomes Project and 44 representative individuals from our studied population. To assess the imputation accuracy, we calculated Dosage-R^2^ (DR^2^), defined as the squared correlation between the estimated allele dose and the true allele dosage. As expected, imputation accuracy showed a positive correlation with minor allele frequency (MAF), saturating at an MAF of approximately 0.05 (Figure S2A–C). After filtering variants with MAF < 0.05 and DR^2^ < 0.8, we retained a total of 10,213,925 autosomal SNPs with an average DR^2^ of 0.93. Additionally, linkage disequilibrium (LD) decay analysis indicated that the average LD did not extend over 100 kb across the genome (Figure S2D). Furthermore, we observed that intron variants and intergenic region variants constituted ∼ 90.36% of sequencing variants, according to genome features (Figure S2E). The sample sizes of 43 body weight-related traits for GWAS analyses ranged from 1047 to 1320 individuals, and the summary information for each phenotype is presented in Table S1.

For RNA sequencing (RNA-seq), we generated 5.28 billion raw reads from 227 muscle samples. After quality control, a total of 1516.95 Gb of high-quality reads were successfully mapped to the ARS-UCD1.2 genome assembly. The average mapping rate was 95.90%, ranging from 92.32% to 97.33% (Table S2). Then, we obtained 16,204 expressed genes for subsequent analyses using a threshold of transcripts per million (TPM) ≥ 0.1 in ≥ 20% of samples. To correct for confounding factors, we considered the top 10 probabilistic estimation of expression residuals (PEER) factors detected by PEER (v1.3) as covariates (Figure S2F). Moreover, we generated single-nucleotide-resolution methylation profiles of 10 muscle samples using whole-genome bisulfite sequencing (WGBS), with an average sequencing amount of 97.16 Gb (ranging from 90.30 Gb to 105.04 Gb). We also performed high-quality assay for transposase-accessible chromatin using sequencing (ATAC-seq) on the same samples, yielding an average of 10.89 Gb of data per sample. The average fraction of reads in peaks (FRiP) score of the 10 samples was 25.81 ± 6.77, suggesting a high-quality ATAC-seq dataset (Table S3) [25]. Additionally, we retrieved and analyzed peak signal data for four histone modifications (H3K4me3, H3K27ac, H3K4me1, and H3K27me3) and CTCF from cattle muscle tissues [46]. Furthermore, we incorporated data from three high-throughput chromosome conformation capture (Hi-C) samples derived from bovine lung tissue [47].

### GWAS analyses based on whole-genome imputed sequence variants

**Figure 1:**
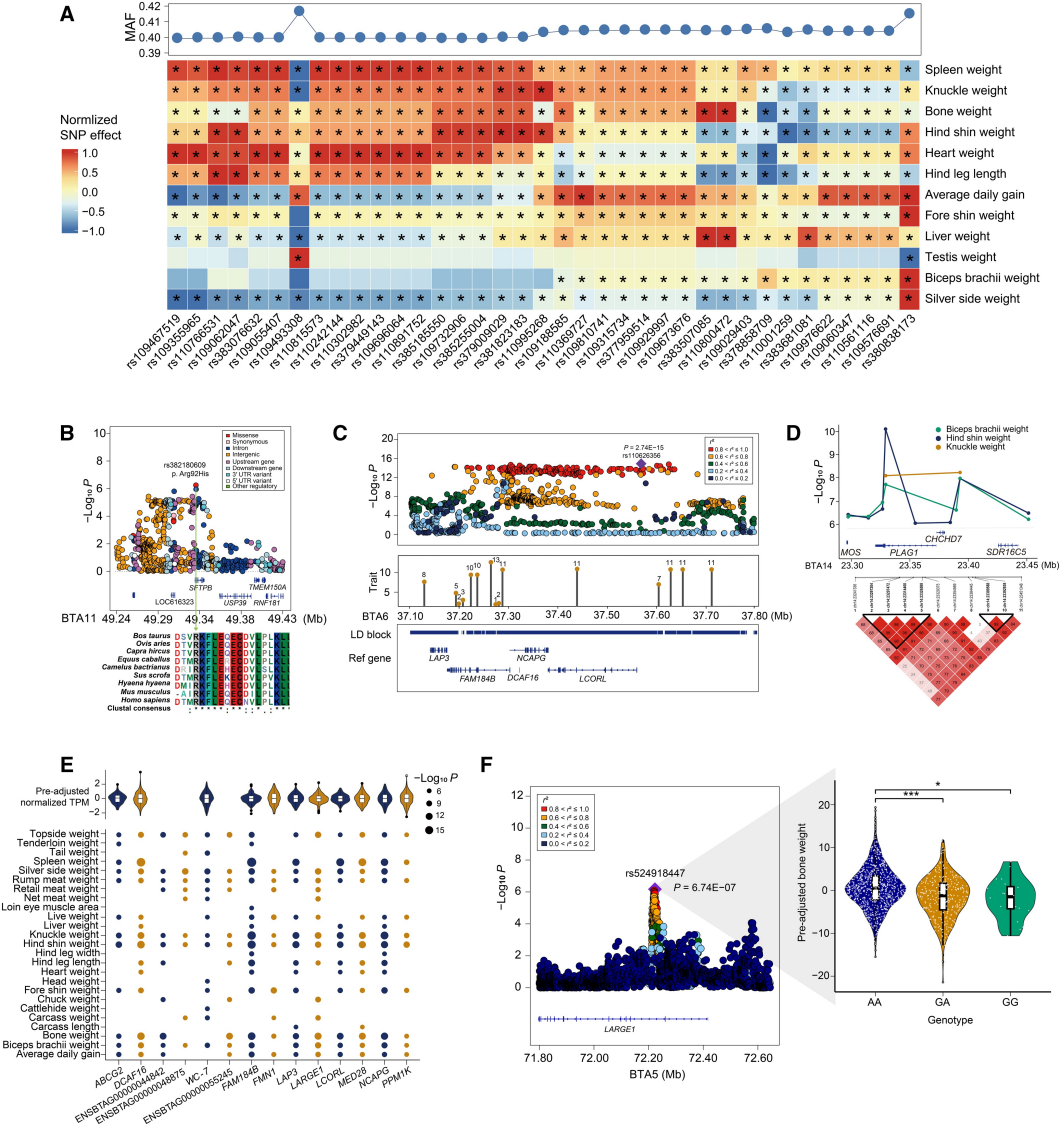
Discovery of important candidate variants and loci for body weight-related traits by multi-strategy GWAS analyses **A**. Heatmap illustrating the effects of 37 SNPs for 12 traits. The color gradient represents the normalized effect of each SNP on the respective trait. “*” in the box indicates the suggestive association (*P* ≤ 1 × 10^−6^). Blue dots on the top panel depict the MAF of variants. **B**. Regional plot of *SFTPB* on BTA11 for fat coverage rate. The top SNP (*P* = 5.68 × 10^−7^) corresponds to the missense variant (rs382180609, p. Arg92His). Different colors indicate various SNP annotations. A multiple sequence alignment of the amino acid sequence encoded by *SFTPB* reveals high conservation across multiple species. **C**. Zoomed-out GWAS plot for a significant candidate variant (*P* = 2.74E−15) associated with spleen weight on BTA6. The lollipop graph displays the number of associated traits for each independent LD block in this region. The third row presents the positions of candidate blocks and genes. **D**. Eleven candidate variants (*P* ≤ 1 × 10^−6^) exhibiting high LD levels for the weights of hind shin, knuckle, and biceps brachii and spanning the *PLAG1*–*CHCHD7* region on BTA14. **E**. Several candidate genes are associated with more than ten traits. The dot size represents the level of significance, and the violin plot illustrates the pre-adjusted expression of these genes. The threshold for gene-based GWAS was set as 1.94 × 10^−6^ (0.05/N). **F**. Regional plot of *LARGE1* on BTA5 for bone weight, with a violin plot indicating the differential genotype effects of rs524918447. GWAS, genome-wide association study; SNP, single nucleotide polymorphism; MAF, minor allele frequency; BTA, *Bos taurus* autosome; LD, linkage disequilibrium; UTR, untranslated region; TPM, transcripts per kilobase million; chr, chromosome.

Our GWAS analyses identified 1078 candidate variant–trait pairs for 14 traits at a genome-wide significance level (*P* < 5 × 10^−8^). These traits included live weight, average daily gain, fat coverage rate, carcass length, hind leg length, and the weights of bone, biceps brachii, cattle hide, fore shin, head, hind shin, knuckle, liver, and spleen. Moreover, 3497 variant–trait pairs were detected for 40 traits at a suggestive significance level (*P* < 1 × 10^−6^) (Figure S3; Table S4). Notably, we observed 725 SNPs with pleiotropic effects, associated with at least two traits. Among these, 37 SNPs (with an average MAF of 0.40) were associated with more than 10 traits (**Figure 1**A).

Specifically, we identified four significant missense variants with strong LD for spleen weight (rs383302973, p. Tyr358Cys; rs382235901, p. Ala154Val), hind shin weight (rs109131933, p. Pro1083Leu), and fat coverage rate (rs382180609, p. Arg92His) (Table S4). We further conducted a conservation analysis for *RP1* and *SFTPB* using multiple sequence alignment. The results revealed that the amino acids affected by these missense variants were highly conserved across species (Figure 1B, Figure S4A).

We detected approximately 370,447 LD-independent regions (Figure S4B) and a total of 265 unique GWAS loci. These loci had a total of 14,775 overlaps with previously reported QTLs for five categories of 203 traits (including health, meat and carcass, exterior, production, milk, and reproduction). Moreover, the QTLs for meat and carcass and production traits accounted for ∼ 64.04% of these overlaps, including metabolic body weight (11.27%) and carcass weight (8.31%) (Figure S4C). Among the 265 GWAS loci, 264 were associated with body-related traits in previous studies, whereas only one novel locus (chr13:81,958,679–81,964,641) was identified for cattle hide weight. In addition, 67 of these 265 loci were associated with more than one trait; 84 and 70 loci were located on *Bos taurus* autosome 6 (BTA6) and BTA14, respectively. We observed a region on BTA6 (37,076,752–37,765,182) that encompassed 11 LD blocks with pleiotropic effects and contained six candidate genes (*LAP3*, *MED28*, *FAM184B*, *NCAPG*, *DCAF16*, and *LCORL*). Within this region, the most significant variant (rs110626356) was detected for spleen weight and exhibited a strong LD pattern with nearby variants (Figure 1C). We also discovered a QTL containing 11 variants linked to the weights of biceps brachii, knuckle, and hind shin. These variants spanned the *PLAG1*–*CHCHD7* locus and displayed a high LD pattern. Among these, one associated SNP (rs134215421) explained 8.21%, 6.93%, and 7.42% of the genetic variance for the three traits, respectively (Figure 1D).

### Identification of candidate genes for weight-related traits using gene-based GWAS analyses

We identified a total of 1522 gene–trait pairs (involving 801 genes associated with 36 body weight-related traits) at a significance threshold of 0.05/N (Figure S3; Table S5). Among these genes, 287 (∼ 35.83%) were associated with at least two traits. The gene-based approach identified 729 candidate genes not detected by the SNP-based GWAS. We also observed that the candidate genes were significantly larger in size than those without significant association (*P* = 1.42 × 10^−17^, two-sided *t*-test), consistent with previous studies in humans [48]. Notably, we identified 14 candidate genes associated with more than 13 traits, indicating their pleiotropic effects on correlated traits (Figure 1E, Figure S4D). Of these, 11 genes were expressed in muscle, and several (including *LAP3*, *DCAF16*, *MED28*, *NCAPG*, *LCORL*, and *PPM1K*) located on BTA6 were also identified in the SNP-based GWAS. In addition, *LARGE1* was detected to be associated with 14 traits in the gene-based GWAS, while this gene exhibited a significant association only with bone weight in the SNP-based GWAS (Figure 1E and F).

### Detection of selective sweeps based on sequence variants

**Figure 2:**
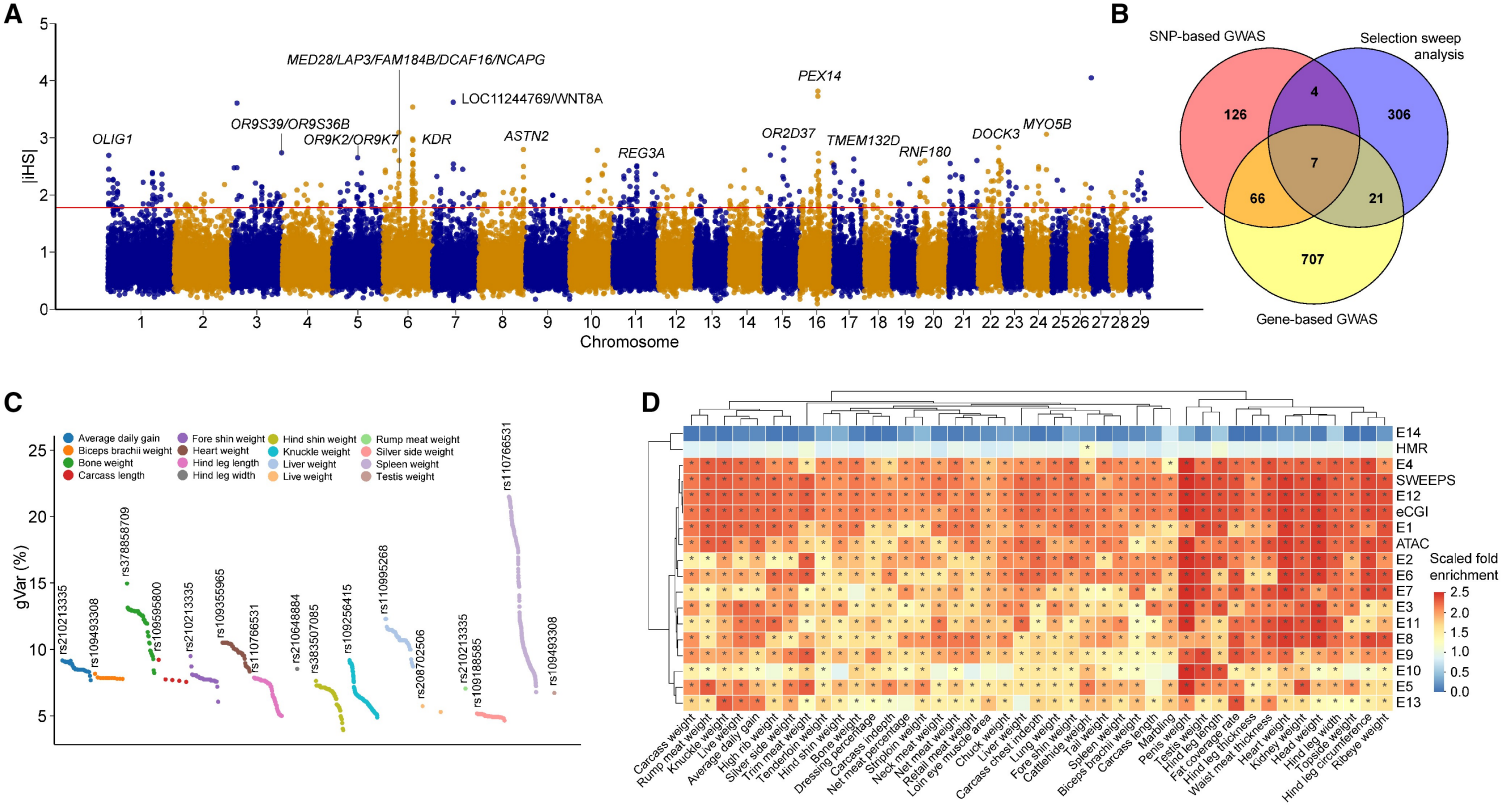
Relationship between GWAS signals and selective sweeps and functional elements **A**. Genome-wide distribution of the top 1% selection signals estimated by iHS. **B**. Venn diagram illustrating the overlap among candidate genes identified by gene-based GWAS, SNP-based GWSA, and selective sweep analyses. **C**. Proportion of genetic variance explained by candidate variants in the target gene for associated traits. Each color represents one trait. **D**. Enrichment analysis of GWAS signals in functional elements and selective sweep regions in cattle. “*” denotes FDR ≤ 0.05. The color scale represents the normalized fold change of GWAS signals within specific functional elements. E1–E14 are categories representing CTCF/active TSS, active TSS, CTCF/promoter, active promoter, flanking TSS, promoter, poised promoter, active enhancer, CTCF/enhancer, primed enhancer, active element, insulator, polycomb-repressed region, and low-signal region. HMR denotes the hypomethylated region detected in muscle tissue; eCGI refers to the experimentally supported CpG island with a methylation level below 30% in at least one sample; ATAC represents the chromatin accessibility region in muscle tissue detected by assay for transposase-accessible chromatin; and SWEEPS denotes the selective sweep region in beef cattle. iHS, integrated haplotype score; TSS, transcription start site; gVar, genetic variance; FDR, false discovery rate.

In this study, we identified a total of 49,410 windows based on the integrated haplotype score (iHS) method (**Figure 2**A). Among these, 494 windows were classified as candidate regions under selection, with their statistical values falling within the top 1% range. Within these regions, we annotated 276 windows corresponding to 338 genes, including *KIT*, *NCAPG*, *MED28*, *LAP3*, and *PEX14*. (Table S6). These genes exhibited significant enrichment in protein binding and positive regulation of multicellular organismal process and cytoplasm, which have been previously associated with traits such as stature, feed efficiency, carcass traits, and coat coloring (Table S7). By integrating SNP-based GWAS, gene-based GWAS, and selective sweep analyses, we identified seven shared genes (*FAM184B*, *DCAF16*, *NCAPG*, *MED28*, *LAP3*, *PCNX2*, and *OR9K7*), and several of them were simultaneously associated with multiple traits (Figure 2B). Notably, five of these candidate genes (*LAP3*, *MED28*, *FAM184B*, *DCAF16*, and *NCAPG*) are located in the *LAP3*–*LCORL* locus. Evaluation of candidate variants within this locus for 16 traits showed that the maximum genetic variance explained by an associated variant ranged from 5.15% to 21.47% of the total genetic variance for the corresponding trait (Figure 2C). In addition, a tag SNP (rs800312260) of *OR9K7*, located within the selective sweep region, was identified as the candidate variant for bone weight, contributing to ∼ 7.12% of the total genetic variance. Conversely, the tag SNP (rs383751136) for *PCNX2* explained ∼ 4.89% of the total genetic variance for the silver side weight.

### GWAS signals are mostly enriched in selection signals and regulatory elements

We observed significant enrichment of GWAS signals in the selective sweep regions [false discovery rate (FDR) ≤ 0.05; Figure 2D], suggesting that genes associated with body weight have likely undergone strong selection during breed formation. To explore the relationship between GWAS signals and regulatory elements, we utilized Genrich to detect consensus open chromatin regions across ten biological replicates in muscle, and obtained a total of 115,890 peaks with an average peak size of 1446.8 bp, covering 6.74% of the autosomes. We identified a total of 98,577 hypomethylated regions (HMRs) by merging WGBS data from ten muscle samples using methpipe (v3.4.3). In addition, we identified a total of 51,222 computer-predicted CpG islands (cCGIs). Among them, 28,302 cCGIs were considered as experimentally supported CpG islands (eCGIs), which have at least five CpG sites detected with more than 5× coverage and a methylation level below 30% in at least one sample [49]. The 14 chromatin states were retrieved from a previous study [46].

Furthermore, we found that GWAS signals exhibited significant enrichment in regulatory regions, including active promoters (E4, average fold enrichment = 2.57), active transcription start sites (TSSs) (E2, average fold enrichment = 2.54), eCGIs (average fold enrichment = 2.39), active enhancers (E8, average fold enrichment = 2.30), and active elements (E11, average fold enrichment = 2.27). In contrast, no significant enrichment was observed in regions with low signal (E14, average fold enrichment = 0.78). We found that GWAS signals for most body weight-related traits showed high enrichment in active TSSs (30.23%) or active promoters (39.53%) (Figure 2D; Table S8). For example, GWAS signals for live weight were enriched in active promoters with a fold enrichment of 2.87 (FDR = 0.003). These results support the notion that GWAS signals in non-coding regions located within the regulatory elements are likely to play a crucial role in the genetic regulation of complex traits.

### Detection of eQTLs for body weight

**Figure 3:**
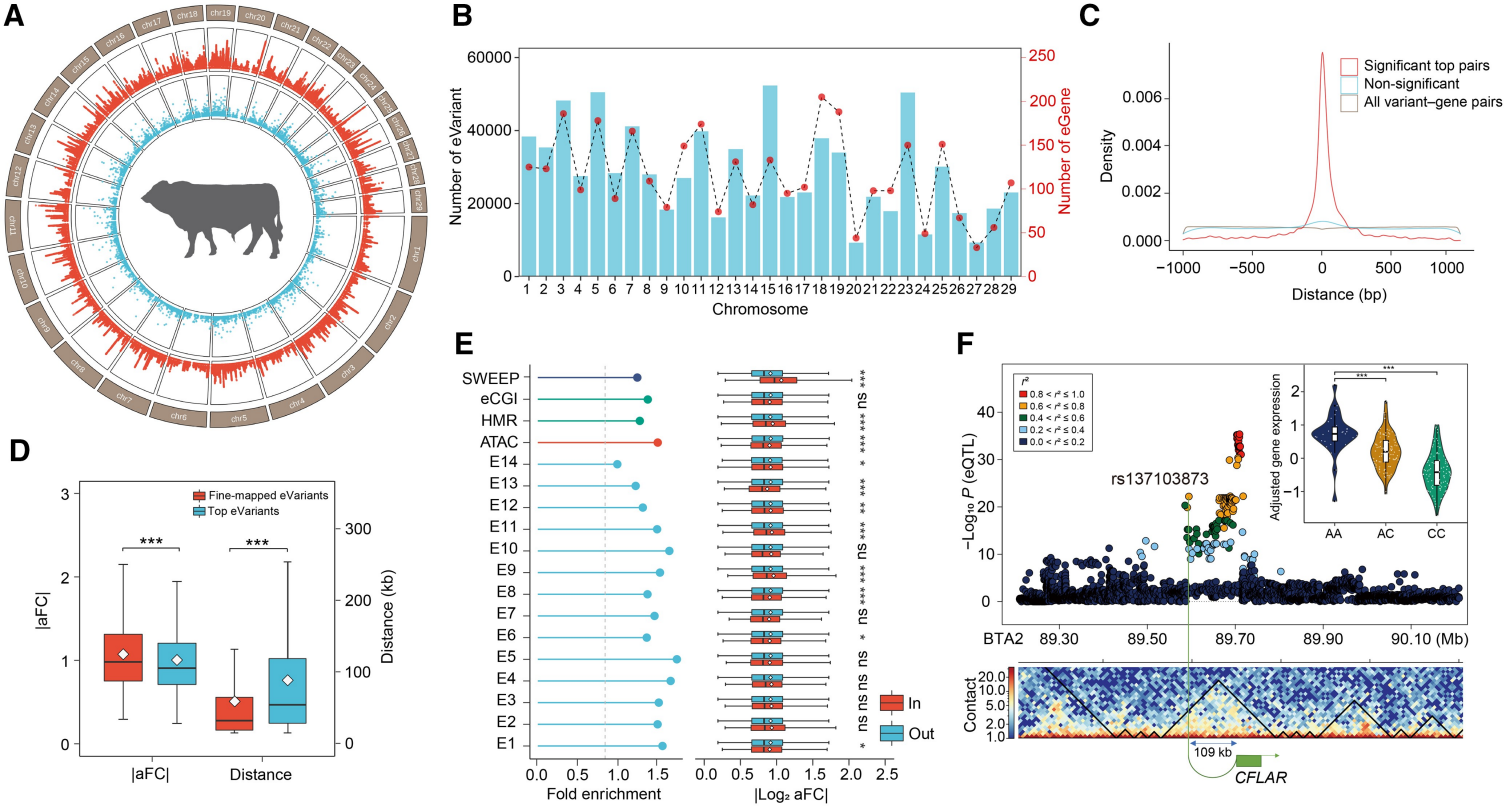
Identification and functional annotation of *cis*-eQTLs in muscle **A**. Circos plot illustrating the distribution of eQTLs (red track) and eGenes (blue track) across the genome. **B**. Numbers of eVariants and eGenes across autosomes. **C**. Density plot depicting the distances between the top eVariant and the TSS of its corresponding eGene. The red, blue, and brown colors indicate the density of eQTL results for significant top pairs, non-significant top pairs, and all variant–gene pairs. **D**. Box plots displaying the distances to the TTS and the aFCs for top eVariants and fine-mapped eVariants. ***, *P* ≤ 0.001 (Student’s *t*-test). **E**. Fold enrichment of eVariants in functional regulatory elements and selective sweep regions, as estimated by genome association test. E1–E14 and other genomic features are described in Figure 2. The box plot on the right shows the comparison of aFC between eVariants within and outside the element. *, *P* ≤ 0.05; **, *P* ≤ 0.01; ***, *P* ≤ 0.001; ns, not significant (Student’s *t*-test). **F**. An example of distant regulation provided by the eGene *CFLAR* and the eVariant (rs137103873) located within the same TAD (BTA2:89,530,000–89,790,000). The individuals with the AA genotype at this locus show higher pre-adjusted TPMs for *CFLAR* than those with other genotypes. ***, *P* ≤ 0.001 (Student’s *t*-test). aFC, allelic fold change; eQTL, expression quantitative trait locus; TAD, topologically associating domain; eGene, expression gene; eVariant, expression variant.

Our analysis identified a total of 3340 significant eGenes with 831,643 unique eVariants, accounting for approximately 20.61% of all expressed genes (16,204) in cattle (**Figure 3**A; Table S9). The largest numbers of eVariants (52,241) and eGenes (205) were observed on BTA15 and BTA18, respectively (Figure 3B). Consistent with previous findings in humans [24] and cattle [27], the significant variants (eVariants) were clustered around the TSSs of genes (Figure 3C). Notably, approximately 44.79% (372,460/831,643) of eVariants were located within 100 kb of the TSSs of their target genes. For the non-eGenes, we observed an enrichment of SNPs with marginally significant *P* values around the TSSs (Figure 3C). Next, we identified 464 eGenes with at least two independent *cis*-eQTLs using deterministic approximation of posteriors (DAP-G) (Table S10). In addition, we found that fine-mapped eVariants had significantly larger effects compared to the top eVariants and were mostly enriched around the TSSs (Figure 3D), which was consistent with previous reports [24,27]. Furthermore, GO enrichment analysis showed that eGenes were significantly enriched in the mitochondrial envelope, oxidoreductase activity, and lipid metabolic processes (Table S11). Finally, we estimated the sharing patterns of muscle *cis*-eQTLs compared with CattleGTEx. We observed that *cis*-eQTLs identified in the current study had a stronger common signal in muscle (π_1_ = 0.49) compared with those from *Bos taurus* (π_1_ = 0.46) in CattleGTEx (Figure S5A).

### Functional annotation of eQTLs

To decipher the functional properties of the identified *cis*-eQTLs, we annotated the associated eVariants based on multi-layer information. We observed that 39.11% and 37.99% of *cis*-eQTLs belonged to intron variants and intergenic region variants, respectively (Figure S5B), while the eVariants were significantly enriched in 3′-UTR, 5′-UTR, and missense variants (Figure S5C). Notably, *cis*-eQTLs annotated as missense variants had larger effect sizes on eGenes than those annotated for other genic features (Figure S5D). By combining chromatin accessibility, HMR, and 14 predicted chromatin states in muscle tissue, we found that *cis*-eQTLs were significantly enriched in active promoters, flanking TSSs, and primed enhancers (Figure 3E). Moreover, the effect sizes of *cis*-eQTLs within functional elements significantly differed from those in other regions. Specifically, *cis*-eQTLs located in active enhancers displayed higher effect sizes than those outside, suggesting their crucial role in gene expression regulation. We also observed enrichment for *cis*-eQTLs within selective sweep regions (Figure 3E). Furthermore, the candidate SNPs from a GWAS of 43 traits were significantly enriched in *cis*-eQTLs (FDR ≤ 0.05, based on 1000 permutation tests). Notably, strong enrichment was observed in the *cis*-eQTLs for average daily gain (Figure S5E). Using Hi-C data from lung tissue, we obtained the topologically associated domains (TADs) and found that ∼ 98.80% of eGenes overlapped with these TADs. Among these eGenes, ∼ 51.17% had their top-eVariants located within the same TADs. Next, we performed 5000 permutation tests using random eGene–SNP pairs based on corresponding distance and observed that the proportion of eGene–eVariant pairs in the same TADs was higher than expected (Figure S5F). For instance, the second *cis*-eQTL (rs137103873) of *CFLAR* was located ∼ 109 kb upstream of the TSS but resided within the same TAD region as *CFLAR*. Remarkably, we observed that individuals with the AA genotype at rs137103873 exhibited significantly higher expression of *CFLAR*, suggesting that gene expression is likely regulated by a distant eVariant through chromatin interactions (Figure 3F).

### Detection of significant gene–trait associations using TWAS analyses

**Figure 4:**
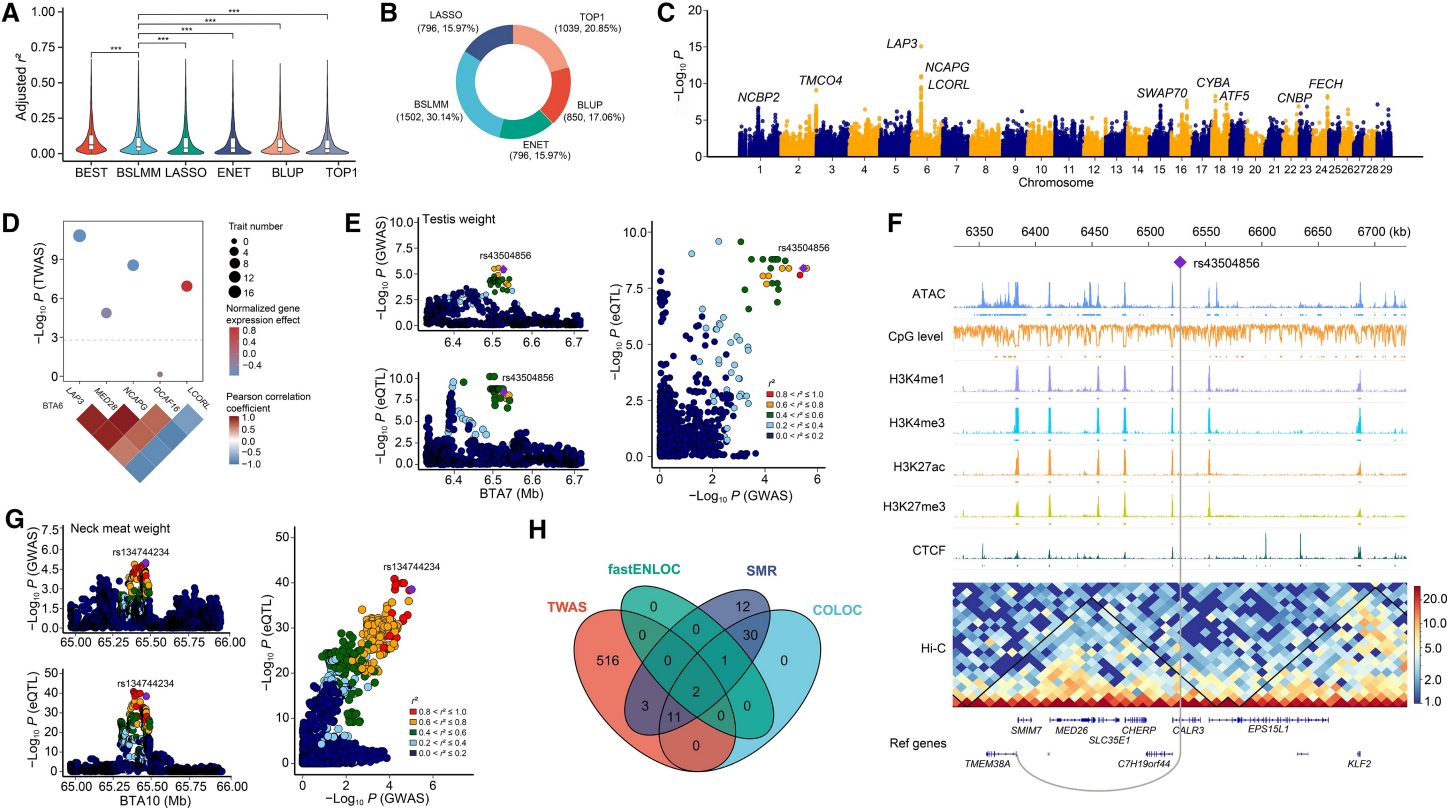
TWAS and colocalization between GWAS loci and eQTLs for body weight-related traits **A**. Prediction accuracy (adjusted *r*^2^) of five models for TPM. **B**. Circular plot illustrating the proportion of genes with the best adjusted *r*^2^ in each model. **C**. Manhattan plot showing TWAS results for 43 traits, with several important candidate genes marked. **D**. Regional plot for five genes on BTA6. Dot size represents the number of traits associated, and dot color represents the normalized gene expression effect on knuckle weight. Heatmap shows the correlations between these genes. **E**. A colocalization example of *TMEM38A* with the highest LCP (0.99) between GWAS signals for testis weight (top left) and *cis*-eQTLs in muscle (bottom left). The scatter plot (right) shows the correlation between the statistical significance of GWAS signals and eQTLs. Dot color represents the LD (*r*^2^) level between the target variant and others. **F**. IGV plot for *TMEM38A*, showing functional annotations from multiple epigenetic marks, the TAD region from lung Hi-C data, and the position of rs43504856 on the genome (the purple rhombus). **G**. The lead GWAS variant (rs134744234) for neck meat weight was associated with the expression of *SORD* (PP.H4 = 0.9957). The panel layout is the same as (E). **H**. Venn diagram showing overlap of genes identified by TWAS, fastENLOC, COLOC, and SMR. IGV, Integrated Genomics Viewer; TWAS, transcriptome-wide association study; LCP, locus-level colocalization probability; fastENLOC, fast enrichment estimation aided colocalization; COLOC, colocalization; SMR, summary-data-based Mendelian randomization; PP.H4, posterior probability for hypothesis 4; BEST, optimal accuracy of each gene among five methods; BSLMM, Bayesian sparse linear mixed model; LASSO, least absolute shrinkage and selection operator; ENET, elastic net regression; BLUP, best linear unbiased prediction; TOP1, single best eQTL; Hi-C, high-throughput chromosome conformation capture.

We estimated the SNP heritability for each expressed gene using the genome-wide complex trait analysis genomic-relatedness-based restricted maximum likelihood (GCTA-GREML) module. A total of 4972 expressed genes were identified with significant non-zero heritability, and the average heritability was 0.169 (Table S12). We evaluated the accuracy of five predictive models for each heritable gene using five-fold cross-validation with actual expression data. Among the five models, the Bayesian sparse linear mixed model (BSLMM) showed significantly higher prediction performance than the others. In fact, the BSLMM model achieved an accuracy of over 30% in predicting heritable genes (**Figure 4**A and B). Next, we selected the optimal model for each gene and imputed the heritable gene expression for the studied population. We identified 1276 significant gene–trait associations (FDR ≤ 0.05) for 29 traits. These associations involved 532 unique candidate genes, of which 47.18% were associated with at least two traits (Figure 4C, Figure S3; Table S13). The genes associated with more than ten traits are presented in Figure S5G. Notably, three genes (*LAP3*, *NCAPG*, and *LCORL*) were detected based on both GWAS and selection signals (Figure 4D). Furthermore, we observed strong positive correlations in the predicted expression of these genes, except for *LCORL*.

### Colocalization analyses pinpoint several important loci

In our study, we conducted colocalization analyses between GWAS loci (*P* ≤ 1 × 10^−5^) and *cis*-eQTLs (FDR ≤ 0.05) using fast enrichment estimation aided colocalization (fastENLOC) and colocalization (COLOC). Using fastENLOC, we identified five genomic regions where GWAS hits for 16 traits colocalized with eQTL signals. Notably, two of these regions including *LAP3* and *NCAPG* exhibited strong colocalization signals. We also found the colocalized *TMEM38A* [locus-level colocalization probability (LCP) = 0.99] between *cis*-eQTLs in muscle and GWAS signals for testis weight. This was captured by a variant (rs43504856) located approximately 145 kb downstream of the TSS of *TMEM38A* (Figure 4E). We found that this variant was located within the same TAD region as *TMEM38A* and its promoter, indicating its potential role in the distant regulation of gene expression (Figure 4F).

Using COLOC, we detected colocalization signals for 78 GWAS loci of 26 traits, involving 44 unique eGenes. Of those, *LAP3*, *MED28*, and *NCAPG* were colocalized with GWAS signals of 16, 17, and 16 traits, respectively, which was consistent with the findings from fastENLOC (Table S14). The association of *TMEM38A* with testis weight was also supported by COLOC [posterior probability of hypothesis 4 (PP.H4) = 0.985]. Interestingly, we found many signals detected only by COLOC. For instance, the lead GWAS variant (rs134744234) for neck meat weight was associated with the expression of *SORD* (PP.H4 = 0.9957) (Figure 4G). Furthermore, GWAS hits for hind shin weight colocalized with eQTL signals of *SLC12A9* and *SPR* (Figure S6A and B). Notably, we found that an eQTL (rs379703744) for *ZNF3* was simultaneously located in GWAS loci for live weight (PP.H4 = 0.9195), average daily gain (PP.H4 = 0.8749), and hind shin weight (PP.H4 = 0.9353) (Figure S6C and D). Additionally, *EHHADH* colocalized with fat coverage rate with PP.H4 = 0.9896 (Figure S6E). A colocalized variant (rs378452852) was observed for the expression of *NDUFA10* and marbling (PP.H4 = 0.92) (Figure S6F).

To assess whether the effect of SNPs on phenotype is mediated through gene expression, we further conducted the summary-data-based Mendelian randomization (SMR) analysis. We obtained 113 significant gene–trait pairs for 29 traits, involving 59 unique eGenes. Most of these genes were identified by COLOC, including *MED28*, *FBXO24*, and *EHHADH*. Finally, we identified only two genes, *LAP3* and *NCAPG*, which were shared by SMR, TWAS, and colocalization analyses (Figure 4H).

### Comprehensive analysis of consensus regions using multi-omics data

To dissect consensus regions with pleiotropic effects and understand their potential genetic regulation, we conducted a multi-trait Chi-square test based on summary statistics across 43 traits. Based on the stringent Bonferroni correction (0.05/10,213,925), we identified two significant loci: the *LAP3*–*LCORL* locus on BTA6 and the *PLAG1*–*CHCHD7* locus on BTA14 (Figure S7A).

**Figure 5:**
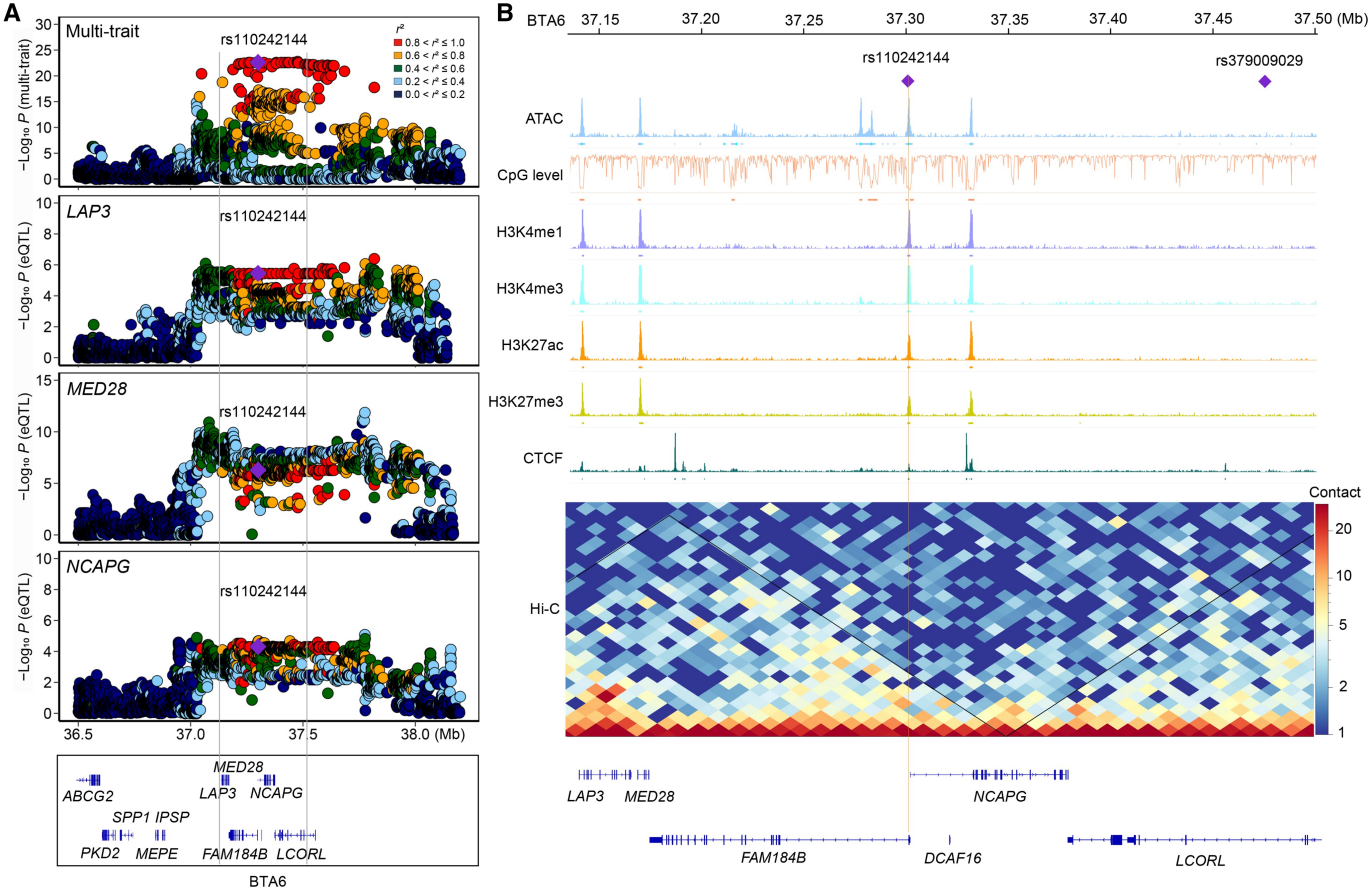
Multi-omics analysis captures the important *LAP3*–*LCORL* locus for body weight-related traits **A**. Regional plots for multi-trait association analysis and eQTL analysis. The zoomed-out scatter plots from top to bottom correspond sequentially to multi-trait GWAS loci and eQTLs (*LAP3*, *MED28*, *NCAPG*) on BTA6. The marked SNP, rs110242144, is colocalized for several traits, including knuckle weight, bone weight, and average daily gain. **B**. Multi-omics plot for the candidate locus on BTA6, showing functional annotations from multiple epigenetic marks. The purple rhombuses mark the positions of two key variants: rs110242144 (the functional variant at this locus) and rs379009029 (the top variant identified by multi-trait GWAS).

**Figure 6:**
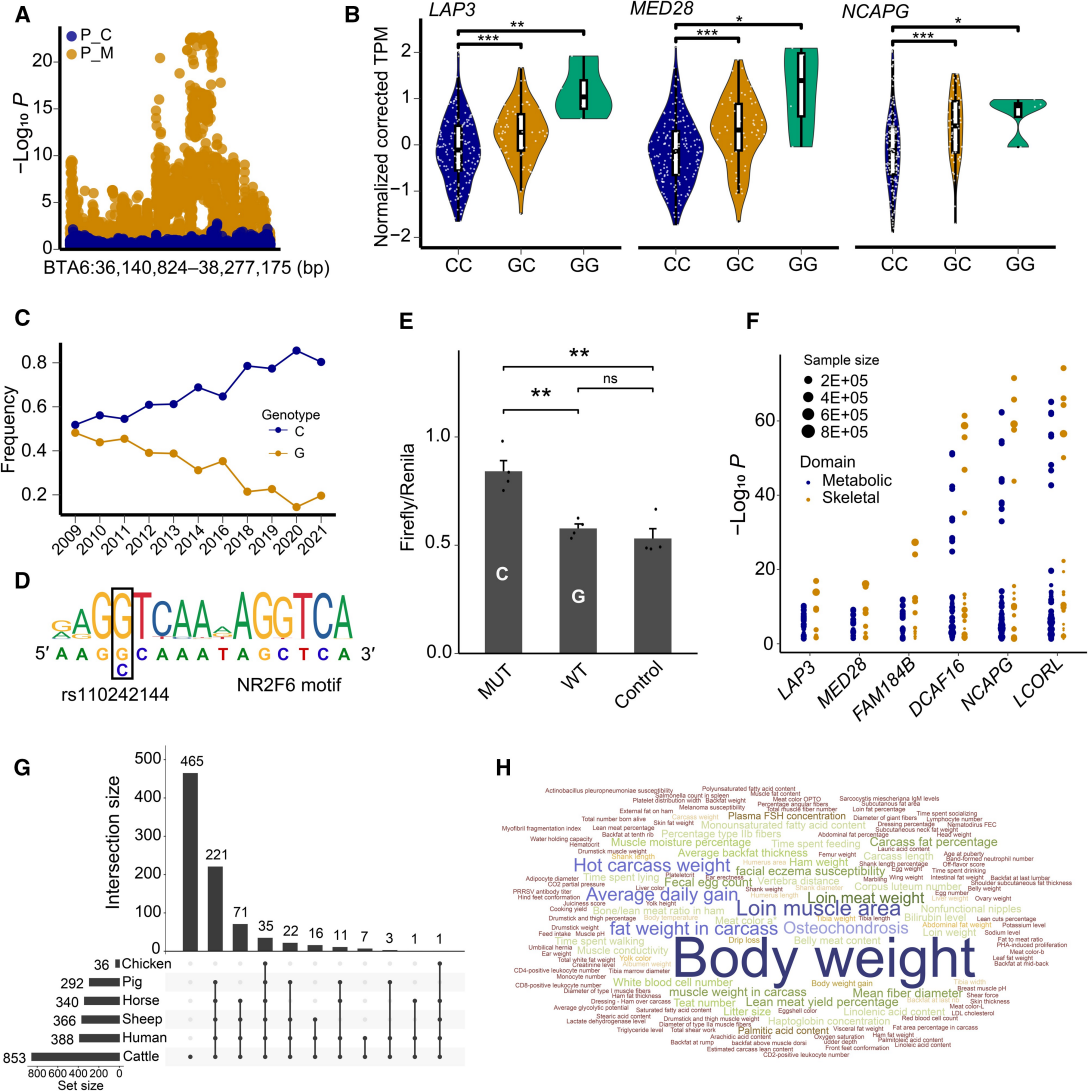
Multi-faceted analysis pinpoints rs110242144 as the potential causal variant in the *LAP3*–*LCORL* locus **A**. Regional plot for the region BTA6:36,140,824–38,277,175 bp. Orange dots denote the −log_10_  *P* values from the multi-trait meta-analysis, and blue dots represent the −log_10_  *P* values from the conditional analysis considering the genotype of rs110242144 as the covariate. **B**. Gene expression levels of *LAP3*, *MED28*, and *NCAPG* in individuals with different genotypes at rs110242144. **C**. Changes in allele frequency (C allele) within the studied population over the past decades. **D**. The allele C of the candidate variant rs110242144 (G>C) may disrupt the NR2F6 motif. **E**. Dual-luciferase reporter assay results. WT stands for wild type, and MUT stands for mutation (rs110242144 G>C). **F**. PheWAS analysis for *LAP3*, *MED28*, *FAM184B*, *DCAF16*, *NCAPG*, and *LCORL*. Blue and orange dots represent the domains of metabolic and skeletal traits, respectively. Dot size is proportional to the sample size of the respective study. **G**. Upset plot showing overlap of candidate variants (from the candidate regions on BTA6 in cattle) across multiple species after conversion by LiftOver. **H**. 3D word cloud displaying traits associated with conserved candidate variants across multiple species. Word size corresponds to trait frequency. In (B and E), significant difference was determined by Student’s *t*-test (*, *P* ≤ 0.05; **, *P* ≤ 0.01; ***, *P* ≤ 0.001; ns, not significant). PheWAS, phenome-wide association study; 3D, three-dimensional.

On BTA6, we identified a total of 853 significant variants with strong LD in the *LAP3*–*LCORL* locus. These variants were associated with six candidate genes including *LAP3*, *MED28*, *FAM184B*, *NCAPG*, *DCAF16*, and *LCORL*. Interestingly, we noticed that a variant (rs110242144) displayed strong LD (*r*^2^ = 0.97) with the top variant (rs379009029) and was significantly associated with the expression of *LAP3*, *MED28*, and *NCAPG*. Moreover, this variant also colocalized with GWAS signals for 14 body weight-related traits (SNP-based GWAS) (**Figure 5**A). Importantly, this variant (rs110242144) was located in an accessible chromatin region near the 5′-end of *NCAPG* and within a DNA methylation valley (*i.e.*, an HMR) (Figure 5B). Analysis of chromatin immunoprecipitation followed by sequencing (ChIP-seq) data for H3K27ac, H3K27me3, H3K4me1, and H3K4me3 in muscle tissue revealed enrichment of these histone marks in this region, alongside open chromatin, which suggests that this region is possibly a bivalent promoter, playing a key role in both positive and negative transcriptional regulation [50]. Moreover, we found that the prioritized variant (rs110242144) was located within the same TAD region as *LAP3*, *MED28*, and *NCAPG*, indicating potential chromatin interactions and distal regulation among these genes (Figure 5B). We further performed conditional analyses for each trait by including rs110242144 as a covariate, and observed that the number of candidate variants significantly decreased in the genomic region at 36.14–38.28 Mb on BTA6 (**Figure 6**A).

Moreover, we observed that individuals carrying the G allele at rs110242144 displayed significantly higher expression of *LAP3*, *MED28*, and *NCAPG* alongside lower phenotypic values, indicating a negative correlation between their expression and trait performance. This finding was consistent with the TWAS results (Figures 4D and 6B). Interestingly, we noted an increasing trend of the C allele at rs110242144 in our studied population (Figure 6C).

Transcription factor (TF) binding motif analysis indicated that the C allele of rs110242144 may disrupt the binding sites for five TFs, including NR2F6, which has been reported as a key regulator of muscle tissue development in previous studies (Figure 6D, Figure S7B). We conducted a dual-luciferase reporter assay to assess the impact of the genetic variation (G>C) at rs110242144 on downstream gene expression. Specifically, we cloned a region spanning from 1881 bp upstream to 295 bp downstream of the TSS of *NCAPG* into the pGL3-Basic vector and replaced the wild-type G allele at position −1667 bp with C (Figure S7C). The activity of the resulting mutant type (C) was significantly higher than that of the wild type (G), indicating the functional effect of this variant (Figure 6E).

On BTA14, the top variant (rs134215421) was also identified using multi-trait association analysis. This variant was located in exon 4 of *PLAG1* and associated with two traits (carcass weight and average daily gain) using SNP-based GWAS (Figure S7D). In this study, although *PLAG1* was not detected in the eQTL mapping in muscle tissue, it was captured for liver and mammary in CattleGTEx [27]. We also found that *TGS1* and *PENK*, two genes located upstream and downstream of *PLAG1*, respectively, colocalized with GWAS signals for knuckle weight and hind shin weight. Notably, we identified a variant (rs133230814) that was associated with hind shin weight and PENK expression, and this variant was located approximately 7 kb downstream of the open chromatin region and HMR within the *PLAG1*–*CHCHD7* locus. This region resided within the same TAD, indicating the complex genetic regulation for the studied traits (Figure S7E).

### PheWAS analysis for the *LAP3*–*LCORL* locus across multiple species

To explore potential associations across species, we first performed a phenome-wide association study (PheWAS) for candidate genes in the *LAP3*–*LCORL* locus (including *LAP3*, *MED28*, *FAM184B*, *DCAF16*, *NCAPG*, and *LCORL*) based on the human GWAS atlas. This analysis unveiled important metabolic and skeletal domains relevant to human stature traits, including standing height, body weight, and body mass index (BMI) (Figure 6F; Table S15). Next, we evaluated the consensus effects of candidate variants on body weight across species. We converted the genomic coordinates of 853 candidate variants identified from multi-trait analyses on BTA6 to GRCh38 (human, 388) by LiftOver, and then to susScr11 (pig, 292), oviAri4 (sheep, 366), EquCab3 (horse, 340), and galGal6 (chicken, 36) as described by a previous study [11] (Figure 6G). Based on the Animal QTL Database (Release 50; 25 April 2023), we observed that the candidate variants on BTA6 were located in QTL regions associated with body weight and carcass weight in farm animals. Meanwhile, these QTLs span many important genes such as *FAM184B*, *MED28*, *LAP3*, *NCAPG*, and *LCORL*, suggesting that these genes may have undergone parallel selection across species (Figure 6H).

## Discussion

GWAS analyses have been widely applied to identify genotype–phenotype associations and candidate genetic variations in various species [15,48,51]. Integrating multi-omics data has emerged as an effective strategy for determining causative variants and understanding the genetic regulation of complex traits [25,26]. In this study, we performed an extensive analysis of 43 body weight-related traits in cattle by integrating genomics, transcriptomics, and epigenomics data. Multiple strategies were employed, including SNP-based GWAS, gene-based GWAS, selective sweep detection, and eQTL mapping. By integrating GWAS signal enrichment analyses for functional elements, as well as colocalization and SMR analyses, we systematically explored the genetic regulation of body weight-related traits and identified genomic regions with functional significance in cattle [26].

Our study highlighted the pleiotropic nature of candidate variants and genes for body weight-related traits using imputed sequencing variants in cattle [15,48]. We found that the pleiotropy was more abundant at the gene level (35.78%) than at the SNP level (20.73%), suggesting that multiple variants within genes can be associated with diverse traits, while the association between genes and traits may be caused by distinct causal variants [48,52]. We also observed that these loci displayed high pleiotropy with strong LD across the genome, suggesting that these regions may harbor potential selection signatures during the breeding process [53–55]. However, testing pleiotropy by the integrated analysis of GWAS, eQTL, and epigenetic data is further required to pinpoint the genes for functional studies [56].

Previous studies in cattle and humans revealed that most complex trait-associated variants were distributed in non-coding regions [30,46,47]. A recent study in cattle also reported that the majority of lead variants in the stature-associated regions were non-coding variants [10]. In the current study, we found that approximately 97.96% of suggestive variants for body weight-related traits were located in non-coding regions, significantly enriched in regulatory elements including promoters, enhancers, and eCGIs in muscle tissue in beef cattle. These results are in line with the findings that GWAS hits for milk production and selective sweeps are highly enriched in chromatin states relevant to active promoters and enhancers in dairy cattle [29,45]. Although these enrichment analyses were performed in different tissue types in different cattle breeds, we observed a consistent enrichment pattern in non-coding regions across the genome. These findings further support that genomic variants associated with complex traits are concentrated in regulatory elements rather than protein-coding regions as reported in humans [57,58].

Our study contributes to extensive transcriptomic as well as multi-layer epigenomic datasets of muscle tissues from the same individuals. These molecular profiles enable an in-depth exploration of the genetic regulation of important traits in beef cattle. Moreover, our data further contribute to important genome resources of farm animals, facilitating comparative analyses with publicly available datasets [26]. In this study, we identified 3340 eGenes in muscle tissue, of which 1194 (35.75%) overlapped with those reported in CattleGTEx (which contains 2766 muscle eGenes), while 2146 were novel eGenes not previously reported in CattleGTEx [27]. These genes were predominantly enriched in metabolic and biosynthetic processes, such as lipid metabolic process and organophosphate biosynthetic process, suggesting their potential roles in the regulation of muscle growth. We believe that the analysis framework and findings of the current study should benefit from the dedicated experimental design, a homogeneous herd, and uniform sampling from the same cattle population [59,60]. Our study expands the functional annotation of the bovine genome and provides insights into regulatory elements for important traits in cattle. The corroborative findings from previous studies indicate that GWAS signals are significantly enriched in functional elements in trait-relevant tissues [28,61]. Importantly, our study reveals that eVariants are significantly enriched in the TSSs of eGenes, GWAS signals, and *cis*-regulatory elements, indicating that functional variants may mediate their regulatory effects through complex patterns across the genome [62,63].

A previous study identified several candidate variations located in the *PLAG1*–*CHCHD7* intergenic region, which were proposed as the most likely *cis*-acting causal variants influencing bidirectional promoter strength [64]. Although no *cis*-eQTL was detected in the *PLAG1*–*CHCHD7* locus, we successfully captured GWAS signals near open chromatin regions associated with knuckle weight and hind shin weight, suggesting potential genetic regulation at this locus. Fu et al. reported that two candidate variants for bovine stature were located in the TSS and promoter regions while exhibiting strong H3K4me3 signals in various tissues [65].

Remarkably, we identified a pleiotropic genomic region on BTA6 with strong evidence by integrating multiple analytical strategies and functional annotations from multi-omics data. Our analysis highlights several prioritized genes affected by functional regulatory elements, including *LAP3*, *MED28*, *NCAPG*, and *LCORL*. These genes have previously been reported to be associated with body weight, size, and growth traits in cattle [10]. Previous analysis also suggests that *LAP3*, *NCAPG*, and *LCORL* show significant correlations between their gene expression and average daily gain in cattle [39,41]. In addition, these genes have been reported to play critical roles in myogenic differentiation in cattle and sheep [66–68]. Moreover, *LAP3* was also identified as a candidate gene for *longissimus dorsi* muscle weight using TWAS [69].

Our study investigated the transcriptional regulation patterns in the *LAP3*–*LCORL* locus associated with body weight-related traits by integrating gene expression, chromatin accessibility, DNA methylation, and other epigenomic data. We identified abundant regulatory elements in this locus, including 24 open chromatin regions and 10 HMRs, which colocalized with histone modification marks with a high frequency. Specifically, we observed that the *LAP3*–*LCORL* region exhibited dynamic changes in chromatin accessibility and DNA methylation, indicating its complex regulatory landscape. These integrative findings may suggest the effect of *cis*-regulatory elements on the transcriptional regulation of weight-related genes [70,71]. By incorporating TAD information, we found intense chromatin interactions among candidate genes including *LAP3*, *MED28*, *FAM184B*, *DCAF16*, and *NCAPG*, implying that enhancer–promoter interactions may be involved in the complex regulation of bovine stature [72]. Further analysis of accurate enhancer–promoter interactions for stature-related genes may leverage higher-resolution three-dimensional chromatin conformation data and more thorough molecular experiments.

In this study, we identified rs110242144 in the *LAP3*–*LCORL* locus as a regulatory variant for body weight-related traits, showing strong pleiotropic effects on both traits and gene expression. This finding may be supported by the higher LD levels and regulatory interactions of TAD between rs110242144 and nearby variants. We hypothesize that this SNP may regulate the *LAP3*–*LCORL* locus through complex regulation patterns, potentially by influencing promoter activity and interaction among TF binding, DNA methylation, and histone modifications. Our dual-luciferase reporter assay further validated the potential role of rs110242144 (located in the promoter of *NCAPG*) in altering the promoter activity of its target gene. In addition, this SNP may also influence the binding of TFs which have been reported as key regulators of feed efficiency in cattle [73].

While this study has significantly enhanced our knowledge of the genetic regulation of body weight, there is potential for further improvement through the inclusion of larger sample sizes, more diverse populations, and analysis of multiple tissues. Further investigations integrating cell catalogs and exploring gene–environment interactions may help systematically elucidate potential molecular mechanisms underlying complex traits [74,75]. Additionally, comprehensive molecular experiments are needed to validate the functions and regulatory mechanisms implicated in this study.

In conclusion, our research provides a comprehensive, multi-omics understanding of the genetic regulation of these candidate regions associated with body weight-related traits in cattle. Moreover, comparative analysis reveals the pleiotropic variants within a promising locus *LAP3*–*LCORL* on BTA6, which exhibit complex regulatory effects on body weight and height. Our findings contribute to unraveling the genetic mechanisms governing weight-related traits and shed valuable light on the genetic improvement of farm animals.

## Materials and methods

### Animals and phenotypes

All animals were derived from Chinese Simmental beef cattle (Huaxi cattle) in Ulgai, Xilingol League, Inner Mongolia Autonomous Region, China. The measurement of various phenotypes was conducted as described in our previous studies [38,76]. A total of 43 body weight-related traits were collected, including live weight, carcass weight, dressing percentage, and net meat weight, according to the GB/T 17238-2022. Detailed information for beef cuts is presented in Table S1. A cohort of 227 cattle, all born between 2017 and 2021 at the Prairie Ulgai, Xilingol League, Inner Mongolia Autonomous Region, China, was transported to a contracted feedlot at an average age of 12 months. All individuals were fed a standard feedlot diet consisting of maize, protein, vitamins, and minerals. After fattening for 12 months, they were transferred to Inner Mongolia Zhongao Food Co., Ltd. for slaughter. Slaughtering and carcass-splitting procedures were implemented in accordance with the national standards. The *longissimus dorsi* muscle samples were immediately collected, rinsed with a sterilized phosphate-buffered saline (PBS) buffer, and frozen in a 2-ml tube for further analysis.

### RNA-seq and expression profiling

Total RNA was extracted from the muscle tissue using TRIzol (Catalog No. 15596026, Invitrogen, Carlsbad, CA) according to the manufacturer’s instructions. Library preparation and sequencing were performed as described in a previous study [77]. After filtering out reads with low quality, a total of 5.05 billion clean reads from 227 samples were obtained. To quantify gene expression, these clean reads were mapped to the ARS-UCD1.2 reference genome using hierarchical indexing for spliced alignment of transcripts 2 (HISAT2) with default setting [78]. In total, 95.90% of the total reads were successfully mapped. Based on the mapped reads, gene features were characterized using Ensembl annotation. StringTie was used to calculate the TPM for each gene among samples [79].

### Genotyping, sequencing, and imputation

All samples were genotyped using the Illumina BovineHD SNP array, with SNP positions determined according to the ARS-UCD1.2 reference genome [80]. A total of 602,053 variants and 1577 individuals were retained after quality control using PLINK (a toolset for whole-genome association and population-based linkage analyses; v1.90) [81] based on the following criteria: call rate > 90%, genotype call rate < 95%, MAF < 0.05, and Hardy–Weinberg equilibrium *P* < 1 × 10^−5^. We then imputed the filtered SNPs on autosomes to the whole-genome sequence level using Beagle 5.4 based on multiple-population panels, which consisted of 1842 individuals from Run8 of the 1000 Bull Genomes Project (ERZ1738264) and 44 representative individuals from our studied population as previously described [82–84].

### Sequencing variant annotation

Quality control for the imputed sequencing variants was performed using thresholds of MAF > 0.05 and DR^2^ > 0.8. A total of 10,213,925 autosomal SNPs with an average DR^2^ of 0.93 were retained for GWAS and eQTL analyses. SnpEff (v5.1) was used to annotate all variants and evaluate their impacts [85]. Functional annotation of SNPs was performed based on gene features from the Ensembl database. These SNPs were grouped into nine functional classes, including intergenic region, missense, synonymous, and intron variants (Table S16).

### Single-SNP-based and gene-based GWAS analyses

In this study, body weight was adjusted using a general linear model. Farm, year, and sex were considered as fixed effects. Body weight before fattening, fattening days, and the first two principal components were included as covariates. The phenotype adjustment and association model for GWAS analysis were described in our previous study [38]. Principal component analysis (PCA) was carried out using genome-wide complex trait analysis (GCTA; v1.93.1) based on imputed whole-genome sequencing (WGS) data from 1577 individuals [86]. The associations between the imputed SNPs and traits were analyzed using mixed linear models in GCTA (v1.93.1). We adopted 5 × 10^−8^ and 1 × 10^−6^ as the significant and suggestive thresholds for the detection of candidate variants, respectively. We estimated the genetic variance explained by candidate variants for the studied traits using the following equation:


(1) $${\;\mathrm{Var}}_{g}(\%)=\;\frac{2p(1-p)\beta^{2}}{\sigma_{a}^{2}}\;*\;100\%$$

where p is the allele frequency of the SNP; β is the allelic substitution effect of the candidate variant for the studied trait; $\sigma_{a}^{2}$ is the additive genetic variance. We also utilized the summary statistics of the GWAS analyses to perform the conditional and joint association analysis (COJO) [86] for the candidate region (on BTA6) by considering the genotype of the target variant (rs110242144) as the covariate.

Gene-based GWAS analysis was performed using multi-marker analysis of genomic annotation (MAGMA; v1.08) [87]. We first annotated all the SNPs according to the genome positions by extending 50 kb upstream and downstream of each gene. A total of 25,804 genes were annotated based on genotype data (10,213,925 variants). To increase the statistical power and sensitivity for different genetic architectures [87], two models (including SNP-wise Mean and SNP-wise Top) were applied for the association test, and genes passing the aggregated *P* value threshold were considered for subsequent analyses. The Bonferroni method with the adjusted threshold (0.05/25,804) was used for multiple testing corrections.

### Multi-trait meta-analysis

To evaluate the pleiotropic effects of identified regions, we conducted a multi-trait association analysis based on Chi-square statistics [88]. The Chi-square statistic for the multi-trait association analysis was calculated for each variant as follows:


(2) $$\chi_{\text{multi}-\text{trait}}^{2}=t_{i}^{\prime }V^{-1}t_{i}$$

where $t_{i}$ is a 43 × 1 vector of the signed *t*-values of variant i for *n* traits, $t_{i}^{\prime }$ (1 × 43) is the transpose of the vector $t_{i}$, and $V^{-1}$ is the inverse of the 43 × 43 correlation matrix. The correlation coefficient was estimated based on the 10,213,925 estimated SNP effects (signed *t*-values) across the traits. The Bonferroni-corrected significance threshold was applied in the multi-trait analyses.

### Selective sweep analysis

The iHS was calculated using the selscan software (v1.2.0a), with a maximum gap size of 800,000 bp between SNPs [89]. The iHS scores were normalized using the norm module in selscan, and single-site values for iHS were averaged in non-overlapping 50-kb windows across the genome. Regions with both the average |iHS| score in the top 1% and more than 10 SNPs were considered candidate regions under positive selection.

### Covariate analysis for eQTL discovery

To avoid false-positive and spurious associations for eQTL mapping [77], gene expression values were normalized for eQTL analyses using the following procedure: (1) genes were selected based on expression thresholds of TPM ≥ 0.1 in ≥ 20% of samples; (2) gene expression values were quantile normalized and then inverse normal transformed across individuals. To adjust for potential batch effects and other technical/biological sources of variation that may affect the transcriptome-wide variation, we estimated potential covariates using the PEER (v1.3) method as described in a previous study [90]. According to the GTEx protocol [23], the PEER factors were recommended to be set at 30. However, the posterior variances of factor weights showed diminishing returns and nearly reached a plateau after 10 PEER factors were included (Figure S2F). Therefore, we used 10 PEER covariates to account for the effects of confounding variables on gene expression in subsequent analyses. To further control the effect of population structure on eQTL discovery, we included the first two principal components.

### eQTL mapping

The *cis*-eQTLs were detected within a 1-Mb window around each gene’s TSS using FastQTL (v2.184) [91]. We calculated empirical *P* values for the associated variant of each gene using the adaptive permutation mode (parameter setting: --permute 1000 10,000). To identify potential eGenes, we determined the lead variant for each gene based on the empirical *P* value, followed by multiple testing correction (FDR ≤ 0.05). The nominal mode in FastQTL was used to calculate the *P* value for each variant–gene pair, and variants with nominal *P* values below the gene-level threshold were considered statistically significant [24,27]. Then, we calculated aFC (defined as the ratio of the expression level of the haplotype carrying the alternative allele to that carrying the reference allele) using the aFC (v0.3) tool [92]. We further applied DAP-G (v1.0.0) to infer multiple independent causal *cis*-eQTLs for each gene [93]. A cutoff of 0.1 (a signal-level posterior inclusion probability greater than 0.9) was set as the inclusion threshold to detect independent eQTLs for target eGenes. To compare the replication rates of *cis*-eQTLs between this study and CattleGTEx, the π_1_ statistic was estimated using the R package qvalue [94].

### TWAS analysis

We conducted a TWAS analysis using functional summary-based imputation (FUSION) [95]. First, we estimated the heritability of each expressed gene using the GCTA software (v1.93.0). Genes with significantly non-zero heritability ($h_{\text{GE}}^{2}$ > 0 and *P* ≤ 0.05) were retained to compute SNP weights for gene expression. Subsequently, we performed five-fold cross-validation using five models: best linear unbiased prediction (BLUP), BSLMM, elastic net regression (ENET), least absolute shrinkage and selection operator (LASSO), and single best eQTL (TOP1). This approach helped us identify the optimal model when the gene’s heritability was significant. The R script make_score.R from FUSION was used to estimate the effects of gene expression, and PLINK (v1.90) was used to predict the expression levels for all 1577 individuals. The association between predicted expression and studied traits was assessed using linear regression, with multiple testing corrections applied via the Benjamini–Hochberg (BH) method to control FDR ≤ 0.05.

### Colocalization and SMR analyses

We performed colocalization analysis for *cis*-eQTLs and GWAS loci using fastENLOC (v2.0) [96]. Briefly, we estimated the probabilistic annotation of *cis*-eQTLs using DAP-G (v1.0.0) and annotated *cis*-eQTLs using the summarize_dap2enloc.pl script. We estimated approximate LD blocks across the genome using PLINK (v1.90) and then calculated the posterior inclusion probability of each LD block using the TORUS tool [97] based on z-scores from GWAS. Using fastENLOC (v2.0) [98], we estimated the LCP for each LD-independent genomic region, and a gene with LCP > 0.8 was considered a significant candidate.

We also conducted colocalization analysis using the COLOC (v5.1.0) method with the function coloc.abf [99]. We estimated PP.H4 and retained the tissue–trait–gene triples with PP.H4 > 0.8. To prioritize target genes and regulatory elements for candidate loci, we next conducted SMR analysis using the SMR software (v1.03) [33]. We used the top-associated eQTL in the *cis*-region as the instrumental variable to test the associations of gene expression with GWAS summary statistics for the 43 traits. In addition, *P*_SMR_ was adjusted by the BH method, and significance was defined as FDR ≤ 0.05.

### Library construction and sequencing for WGBS

Genomic DNA from ten *longissimus dorsi* muscle samples was extracted using the QIAamp DNA Mini Kit (Catalog No. 51304, QIAGEN, Valencia, CA) according to the manufacturer’s instructions. Genomic DNA was sheared to fragments of 200–300 bp using a Covaris S220 (Covaris, Woburn, MA), followed by terminal repair and adapter ligation. Bisulfite conversion was performed using the EZ DNA Methylation Gold Kit (Catalog No. D5005, Zymo Research, Irvine, CA). After processing, the unmethylated C became U [which became T after polymerase chain reaction (PCR) amplification], while the methylated C remained unchanged. The WGBS library for each sample was constructed by PCR amplification. The integrity, concentration, and size of DNA fragments of each library were measured using an Agilent 2100 Bioanalyzer (Agilent Technologies, Santa Clara, CA). Library quantification was performed using iCycler quantitative PCR machine (Bio-Rad, Hercules, CA). Finally, the WGBS libraries were sequenced on the Illumina HiSeq X Ten platform to generate 150-bp paired-end reads by Novogene (Novogene, Beijing, China).

### Identification of methylcytosines, CpG islands, and HMRs

The raw WGBS data were filtered using the fastp software (v0.21.0) [100]. The filtration criteria were as follows: (1) remove adapter sequences; (2) discard reads with N content exceeding 10% of the read bases; and (3) remove reads with low-quality bases exceeding 50% of the read bases. The index of the ARS-UCD1.2 reference genome was built using bismark_genome_preparation in Bismark (v0.23.0) [101]. The clean reads from each sample were aligned to the reference genome using Bismark (v0.23.0) [101] with default parameters. After removing the duplicate reads, the methylcytosine information was extracted using bismark_methylation_extractor and summarized using bismark2summary.

Only CpG sites with at least 10× coverage were used for CGI and HMR detection. For CGI detection, we used the CpGIScan software with default parameters (length ≥ 500 bp, GC content ≥ 55%, and ObsCpG/ExpCpG ratio ≥ 0.65) on the cattle reference genome [102]. The methylation level of each cCGI for each sample was calculated, and the eCGI was defined as having a methylation level below 0.3 in at least one sample. For HMR detection, we chose a 10-kb window size for muscle samples using methpipe (v3.4.3) with default parameters [103].

### Library construction and sequencing for ATAC-seq

ATAC-seq libraries were constructed from ten cryopreserved muscle tissue samples according to the protocol described by Halstead and his colleagues [104]. In brief, frozen tissue was chopped, resuspended in homogenization buffer, and ground into a homogeneous suspension, followed by filtering with a cell strainer. Transposition reactions were performed using the Illumina Nextera DNA Library Preparation Kit [Catalog No. FC-121-1030, Illumina, San Diego, CA; containing the transposase MIX and two equimolar adapters (adapter 1 and adapter 2)] at 37°C for 30 min. Transposed DNA was then purified with the MinElute PCR Purification Kit (Catalog No. 28004, QIAGEN). Eluted DNA was amplified with custom-synthesized index primers using real-time PCR. After PCR amplification, libraries were purified with the Agencourt AMPure beads (Catalog No. A63880, Beckman Coulter, Brea, CA) and quantified by Qubit (Catalog No. Q33231, Thermo Fisher Scientific, Waltham, MA). The clustering of the indexed samples was performed on a cBot Cluster Generation System using the TruSeq PE Cluster Kit v3-cBot-HS (Catalog No. PE-401-3001, Illumina) according to the manufacturer’s instructions. After cluster generation, the libraries were sequenced on the Illumina Hiseq X Ten platform to generate 150-bp paired-end reads.

### Mapping and peak calling for ATAC-seq

Raw sequencing reads were initially quality-assessed by FastQC (v0.11.5). Subsequently, raw reads were filtered using fastp (v0.21.0) [100] with the following strict criteria: (1) remove reads with more than 40% of the read bases having a base quality score below 15; (2) discard reads with more than 6 N bases; (3) remove adapter sequences; and (4) discard reads shorter than 50 bp after trimming. Clean reads were aligned to the ARS-UCD1.2 reference genome using Bowtie 2 (v2.2.7) [105] with default settings. Binary alignment map (BAM) files were sorted using SAMtools (v1.9) [106]. Peak calling for muscle samples was performed using Genrich (RRID:SCR_025320) with the following parameters: -m 30, -j (ATAC-seq mode), -r (to remove PCR duplicates), -e MT (to exclude mitochondrial chromosomes), -q 0.05 (FDR-adjusted *P* value threshold).

### ChIP-seq and Hi-C analyses

ChIP-seq data were obtained from the Gene Expression Omnibus (GEO: GSE158430) [46], which included muscle samples from two Hereford cattle profiled for H3K4me3, H3K4me1, H3K27ac, H3K27me3, and CTCF. We also downloaded the 14 distinct chromatin states of muscle tissue predicted by ChromHMM. Subsequent analysis focused on narrow peaks to investigate the characteristics and biological functions of these epigenetic signatures according to a previous study by Kern and his colleagues [46].

Hi-C data from bovine lung tissue were retrieved from the National Center for Biotechnology Information (NCBI) Sequence Read Archive (SRA: SRR5753600, SRR5753603, and SRR5753606) [47]. Trim Galore (v0.6.8) was used to remove adapter sequences and low-quality reads with default settings. Cleaned Hi-C reads were mapped to the ARS-UCD1.2 reference genome using bwa (v0.7.17). A Hi-C contact matrix was built with a resolution of 10 kb using the hicFindTADs module in HiCExplorer (v3.7.2) [107]. TADs were identified in the Hi-C data based on multiple testing corrections (FDR ≤ 0.01).

### GWAS signal and eQTL enrichment analyses

SumGSE was used to perform GWAS signal enrichment analysis [108]. The enrichment patterns of eQTLs within functional elements and gene features were analyzed using genomic association tester (GAT; v1.3.6) [109] and TORUS [97]. Gene set enrichment analysis was conducted using clusterProfiler in R and g:Profiler [110,111].

### PheWAS analysis and QTL comparison for candidate variants

Protein sequences of the target genes were downloaded from the NCBI Protein database. Multiple sequence alignment was performed using BioEdit (v7.2.6.1) to evaluate protein sequence conservation. The disruptive effects of candidate SNPs on TF binding sites (TFBSs) were analyzed using Motifbreak R (v2.2.0) combined with the JASPAR2022 database [112]. The significant variants on BTA6 were lifted to the corresponding positions in the genome assemblies of pig (susScr11), horse (EquCab3), chicken (galGal6), sheep (oviAri4), and human (GRCh38) [80]. PheWAS analysis was conducted using the GWAS ATLAS database [48]. The associated traits for the studied variants or genes were queried in the PheWAS module and sorted by domain and *P* value. Traits with a *P* value less than the Bonferroni-corrected significance threshold (0.05/number of traits) were considered candidate traits. QTL information on farm animals was retrieved from the Animal QTL Database (Release 50). The comparative analyses between variants and QTLs were performed by BEDTools [113].

### Cell culture and plasmid construction

HEK293T cells were cultured in growth medium (90% Dulbecco’s Modified Eagle Medium–High Glucose, 10% fetal bovine serum, 100 μg/ml streptomycin, and 100 g/ml penicillin) according to our previous study [114]. The incubator was set to provide a humidified atmosphere of 37°C and 5% CO_2_.

A fragment of the *NCAPG* promoter was amplified from bovine DNA using the 2× Phanta Max Master Mix (Dye Plus) (Catalog No. P525-02, Vazyme, Nanjing, China) following the manufacturer’s instructions. The primers used were *NCAPG*-F-*Kpn*I (5′-GGAGGTACCAGGCAGCTAGTGCCCAATTT-3′) and *NCAPG*-R-*Mlu*I (5′-CGTACGCGTTGCTTCCTTTCCCACACCTG-3′), and the annealing temperature was set to 64°C. After amplification, the purified PCR fragments were digested with *Kpn*I and *Mlu*I (Catalog Nos. 1068A and 1071A, TAKARA, Kusatsu, Japan) at 37°C for 1 h, and then purified using the E.Z.N.A. Gel Extraction Kit (Catalog No. D2500-01, Omega, Norcross, GA). The purified fragments were ligated into the pGL3 vector using T4 DNA ligase (Catalog No. C301-02, Vazyme) and transformed into competent cells (Catalog No. C502-02, Vazyme). The rs110242144 G>C mutation was introduced using the Mut Express MultiS Fast Mutagenesis Kit V2 (Catalog No. C215-02, Vazyme) with the primers *NCAPG* Mut-F (5′-ACTTCAAGcCAAATAGCTCAATTCAGGCACTGG-3′) and *NCAPG* Mut-R (5′-GCTATTTGgCTTGAAGTTTCCTTTTGATCTCGTAA-3′). Plasmid DNA was extracted from 16-h bacterial cultures using the EndoFree Mini Plasmid Kit II (Catalog No. 4992422, Tiangen, Beijing, China) and verified by Sanger sequencing at Sangon Biotech (Beijing, China). The resulting constructs were named pGL-WT and pGL-MUT, respectively.

### Dual-luciferase reporter assay

Prior to transfection, HEK293T cells were seeded into a 24-well plate and incubated until they reached approximately 80% confluency. Each well was then transfected with 200 ng of the recombinant vector (pGL3, pGL-WT, or pGL-MUT), along with 100 ng of pGL4.74 hRluc/Tk, using Lipofectamine 3000 Transfection Reagent (Catalog No. L3000150, Invitrogen). After 48 h, cell lysates were collected and used to measure the relative transcriptional activity of each group using the Dual-Glo Luciferase-Assay System (Catalog No. E2920, Promega, Madison, WI), following the manufacturer’s instructions. The relative luciferase activities were assessed on an Infinite 200 PRO (TECAN, AG, Switzerland). All experiments were carried out in parallel and in quadruplicate. Statistical difference in relative luciferase activity between the two groups were evaluated using Student’s *t*-test implemented in the R package ggsignif.

## Ethical statement

All animal procedures were performed strictly according to the guidelines proposed by the China Council on Animal Care and in compliance with the Animal Research: Reporting *In Vivo* Experiments (ARRIVE) guidelines. Tissue samples from beef cattle were collected with the approval of the Ethics Committee of Science Research Department of the Institute of Animal Science, Chinese Academy of Agricultural Sciences (Approval No. IAS2020-48).

## Supplementary Material

qzaf067_Supplementary_Data

## Data Availability

The WGBS, ATAC-seq, and RNA-seq datasets have been deposited in the Genome Sequence Archive [115] at the National Genomics Data Center (NGDC), China National Center for Bioinformation (CNCB) (GSA: CRA011244; BioProject: PRJCA017824 and PRJCA017331), and are publicly accessible at https://ngdc.cncb.ac.cn/gsa.

## CRediT author statement

**Qunhao Niu:** Conceptualization, Software, Formal analysis, Investigation, Writing – original draft. **Jiayuan Wu:** Software. **Tianyi Wu:** Validation. **Tianliu Zhang:** Investigation. **Tianzhen Wang:** Resources. **Xu Zheng:** Resources. **Zhida Zhao:** Software. **Ling Xu:** Methodology. **Zezhao Wang:** Data curation. **Bo Zhu:** Data curation. **Lupei Zhang:** Resources. **Huijiang Gao:** Resources. **George E. Liu:** Writing – review & editing, Funding acquisition. **Junya Li:** Funding acquisition, Project administration. **Lingyang Xu:** Methodology, Writing – review & editing, Supervision, Funding acquisition. All authors have read and approved the final manuscript.
