# Supplementary material for: Comprehensive Multi-omics Analysis of Regulatory Variants for Body Weight in Cattle
Source: Genomics Proteomics Bioinformatics. 2025 Aug 18;23(4):qzaf067. doi: 10.1093/gpbjnl/qzaf067 (PMC12701805; doi:10.1093/gpbjnl/qzaf067)
Supplement: qzaf067_Supplementary_Data [file qzaf067_supplementary_data.zip › Supplementary material captions.docx]

# Supplementary material

**Figure 1** **A workflow for comprehensive multi-omics analysis of regulatory variants for body weight in cattle**

To investigate traits related to body weight using sequencing variants in cattle, our study performed a multi-faceted analysis includes selection sweeps, GWAS, TWAS, eQTL mapping, colocalization, SMR, multi-omics function annotation, evolutionary analysis, and molecular experiment validations. WGS, whole-genome sequencing; RNA-seq, RNA sequencing.

**Figure 2**  **The basic statistic information of 10,213,925 imputed whole genome variants**

**A.** The imputation accuracy (DR^2^) varies with the MAFs on BTA1 (left) and BTA29 (right). The black line is the average accuracy. **B.** The density profile of imputed SNPs across the genome. **C.** A boxplot of MAF across 29 autosomes. **D.** The plot of LD decay across the genome. **E.** A pie plot shows the classification of SNP annotation for 10,213,925 variants. **F.** A diagnostic plot of the factor relevance from PEER, indicating that factor weight variances decrease to zero when the inferred hidden PEER factors reach 10. PEER, probabilistic estimation of expression residuals; DR^2^, Dosage-R^2^.

**Figure 3 The landscape of genetic association between 43 body weight related traits and variant, gene, and gene expression**

Association maps of genetic analyses including GWAS, gene-based GWAS, and TWAS. The rows and columns show the trait name and chromosomes. The red dots represent the candidate genes in gene-based GWAS, the light-blue triangles represent the candidate variants in GWAS, and the green squares represent the candidate genes in TWAS.

**Figure 4 Example of GWAS signals and comparation of GWAS loci revealed in current study and Cattle QTL Database**

**A.** The regional plot of *RP1* on BTA14 for hind shin weight. A candidate SNP is the missense variant (rs109131933, p. Pro1083Leu). The different colors represent the various classes of annotation for SNPs. It also includes haplotype LD analyses near rs109131933 on the candidate gene *RP1*. Multiple sequence alignment of the amino acid sequence encoded by the *RP1* gene showed that p. Pro1083Leu has high conservation across different species. **B.** The distribution of LD-independent regions across the genome. The red dots represent the mean length of blocks for each chromosome. **C.** Percentage of QTL type (pie chart), trait related to production and meat and carcass QTLs (barplots) for the QTL annotation results obtained for GWAS loci revealed in this study. **D.** A heatmap showing genetic correlations between 43 traits.

**Figure 5** **Functional characterization of *cis*-eQTLs in muscle**

**A.** Replication of muscle *cis*-eQTLs between the current study and CattleGTEx. Rows are the discovery population and columns are the validation population. Muscle_indicus, Muscle_ taurus, Muscle_ cross, and Muscle represent *cis*-eQTL in CattleGTEx from *Bos indicus*, *Bos taurus*, their crosses, and all of them. Muscle_CSC represents the muscle eQTL discovered in this study. **B.** The proportion barplot shows the annotation of eVariants. **C.** The estimated enrichment (odds ratio) of eVariants in SNP annotation from SnpEff by the TORUS software. **D.** A boxplot comparing aFC of eVariants with different annotation classifications. **E.** c*is*-eQTLs (*P* = 0.001) in muscle exhibit significantly higher enrichment for the top 10% of SNPs (red) associated with average daily gain compared with genome-wide SNPs (blue). **F.** The observed percentages of eGene–eVariant pairs within the same TADs are significantly higher (FDR < 0.01, one-sided) than expected in muscle. **G.** An upset plot displaying candidate genes from TWAS for the number of associated traits.

**Figure 6** **The example for colocalized signals between cis-eQTLs in muscle and GWAS signals**

**A.–F.** The colocalized signal from COLOC for hind shin (A and B), liveweight (C), average daily gain (D), fat coverage ratio (E), and marbling (F). The dot color represents the LD level (r^2^) between the shared variant and nearby variants. The left panels are the regional plots for GWAS signals. The right panels are the regional plots for *cis*-eQTL in muscle. Middle panels are the scatter plots for the significant correlation between GWAS and eQTL. The dot color represents the LD level (r^2^) between the target variant and others.

**Figure 7** **Functional annotation and analyses for key QTLs of body weight related traits**

**A.** The Manhattan plot displays the results of the multi-trait meta-analysis for 43 traits. Two prominent candidate loci are highlighted on the plot. **B.** Analysis of transcription factor motif disruption for the variant rs110242144 reveals five potential candidate motifs in the JASPAR2022 database. **C.** Sequencing results for the pGL-WT and pGL-MUT vectors, with the red block indicating the mutation site (rs110242144 G>C). **D.** Regional plots for BTA14, showing the shared variant (rs133230814) that is associated with GWAS for hind shin and eQTLs for *PENK*. The dot colors represent the LD levels (r^2^) of SNPs with the top variant. **E.** The multi-omics plot for the candidate locus on BTA14 displays functional annotations from various epigenetic marks, and the purple rhombi indicates the position of the target variants on the genome.

**Table S1 The information of the GWAS traits**

**Table S2 Sample information for the RNA sequencing**

**Table S3** **The information of summary statistics for the WGBS and ATAC-seq**

**Table S4** **Candidate variants revealed by single-trait GWAS for 43 body weight related traits**

**Table S5** **Candidate genes revealed by gene-based GWAS for 43 body weight related traits**

**Table S6 Putative selected genes revealed by selective sweeps using iHS statistics in Huaxi cattle**

**Table S7** **GO enrichments for candidate selected genes revealed by selective sweeps**

**Table S8**  **The GWAS signal enrichment analyses for functional elements and selective sweeps**

**Table S9**  **The candidate expressed genes revealed by fastQTL in muscle**

**Table S10**  **The fine-mapping results for the candidate eQTL in muscle**

**Table S11**  **The GO enrichments for the eGenes in cattle muscle**

**Table S12** **The predictive performance across the five methods for the significant heritable eGenes**

**Table S13**  **The candidate eGenes for body weight traits revealed by TWAS**

**Table S14 The candidate colocalized genes between GWAS and eQTL revealed by COLOC**

**Table S15 The PheWAS results for six candidate genes of body weight in human GWAS atlas database**

**Table S16 The annotation classification for the variants in the iWGS panel**
