# Supplementary figures and images for: Comprehensive Multi-omics Analysis of Regulatory Variants for Body Weight in Cattle
Source: Genomics Proteomics Bioinformatics. 2025 Aug 18;23(4):qzaf067. doi: 10.1093/gpbjnl/qzaf067 (PMC12701805; doi:10.1093/gpbjnl/qzaf067)

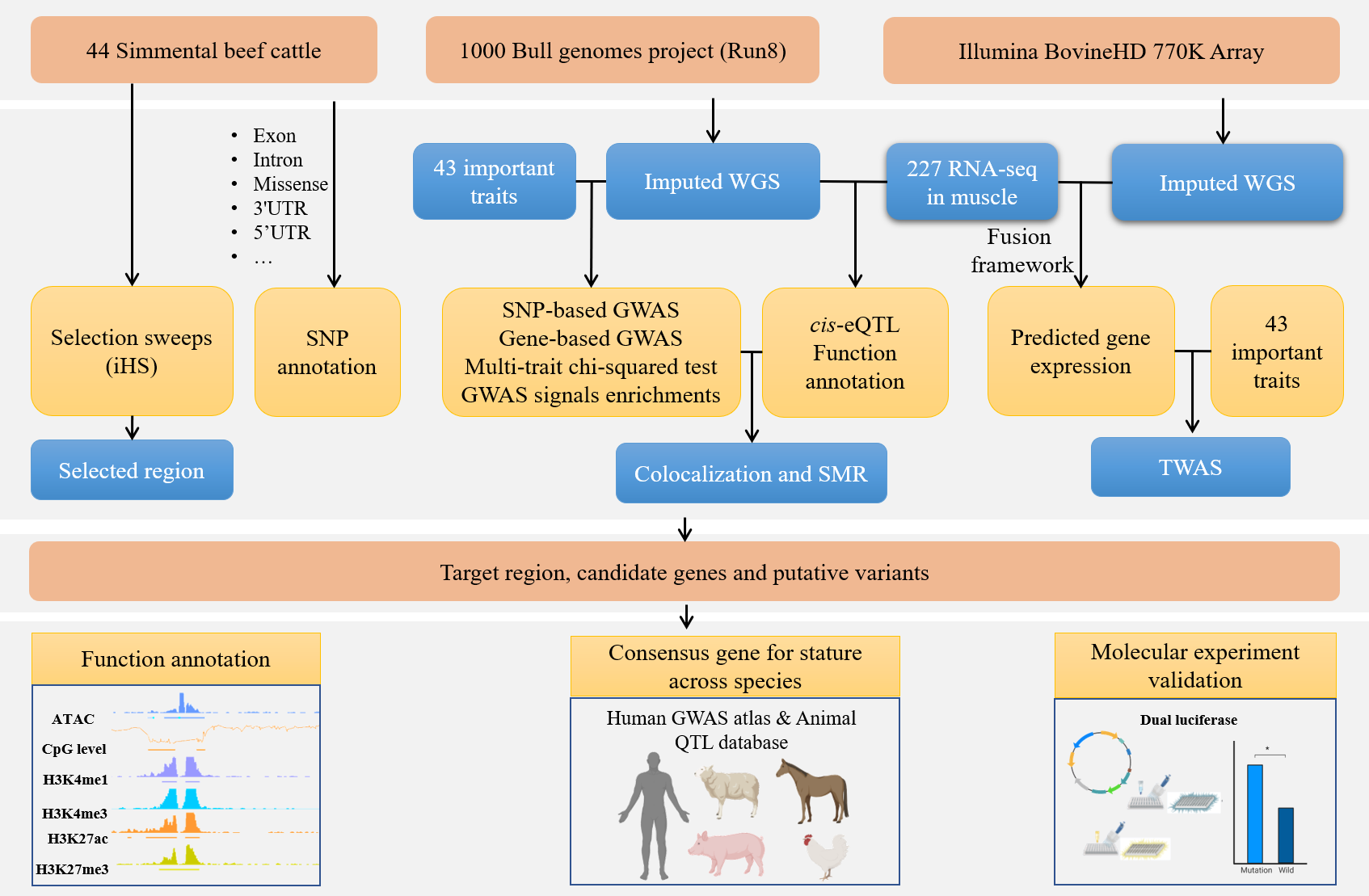

Supplement: qzaf067_Supplementary_Data [file qzaf067_supplementary_data.zip › Figure S1.png]

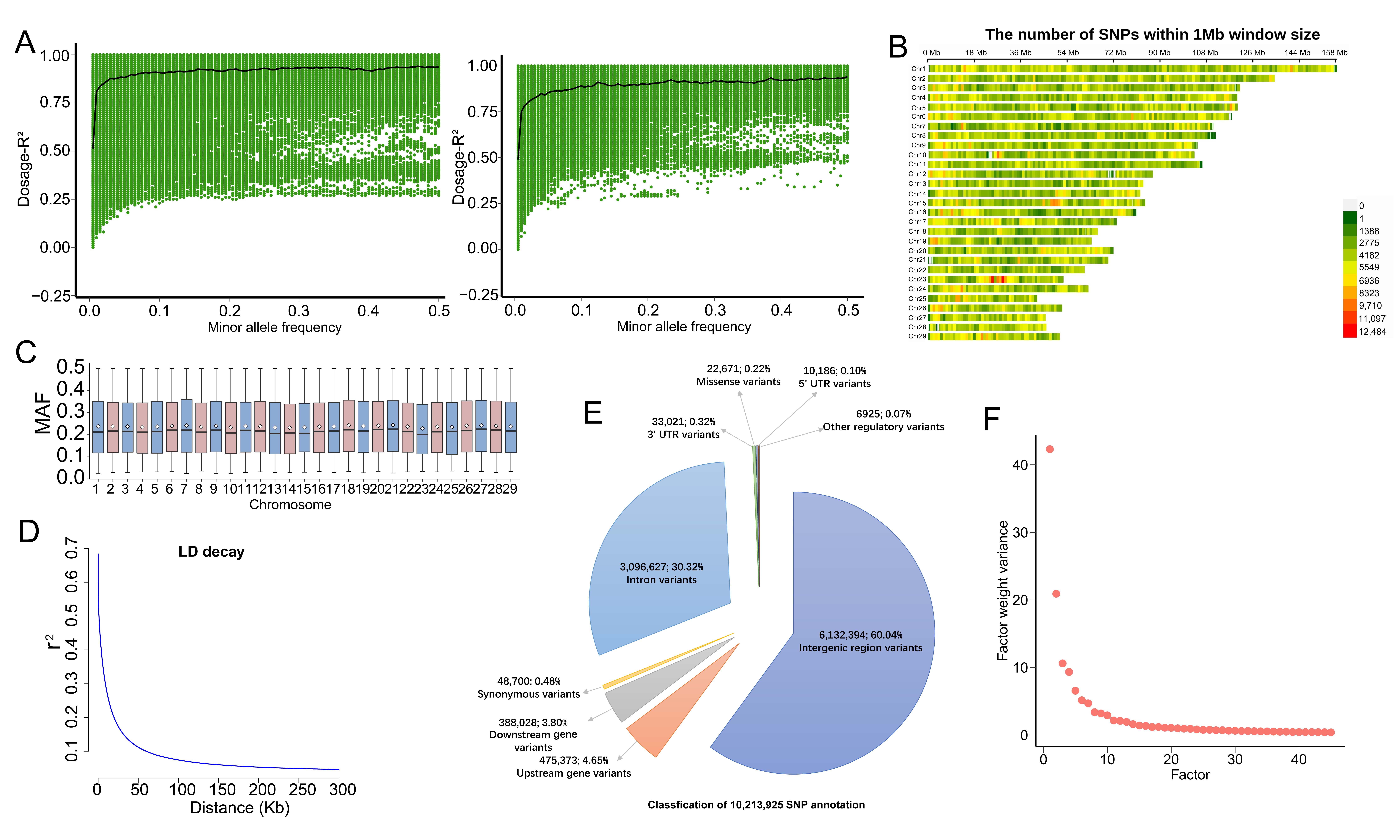

Supplement: qzaf067_Supplementary_Data [file qzaf067_supplementary_data.zip › Figure S2.jpg]

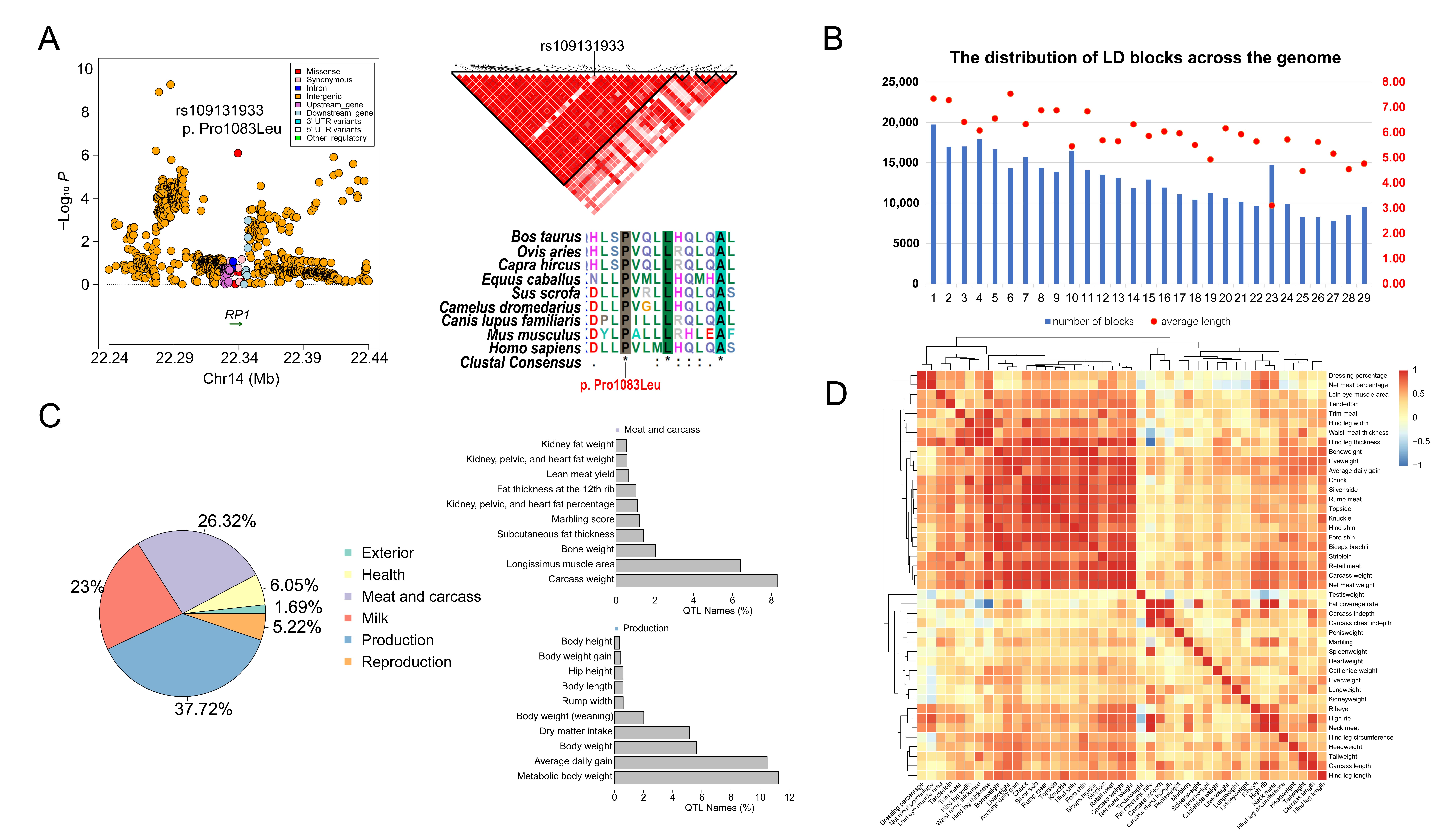

Supplement: qzaf067_Supplementary_Data [file qzaf067_supplementary_data.zip › Figure S4.jpg]

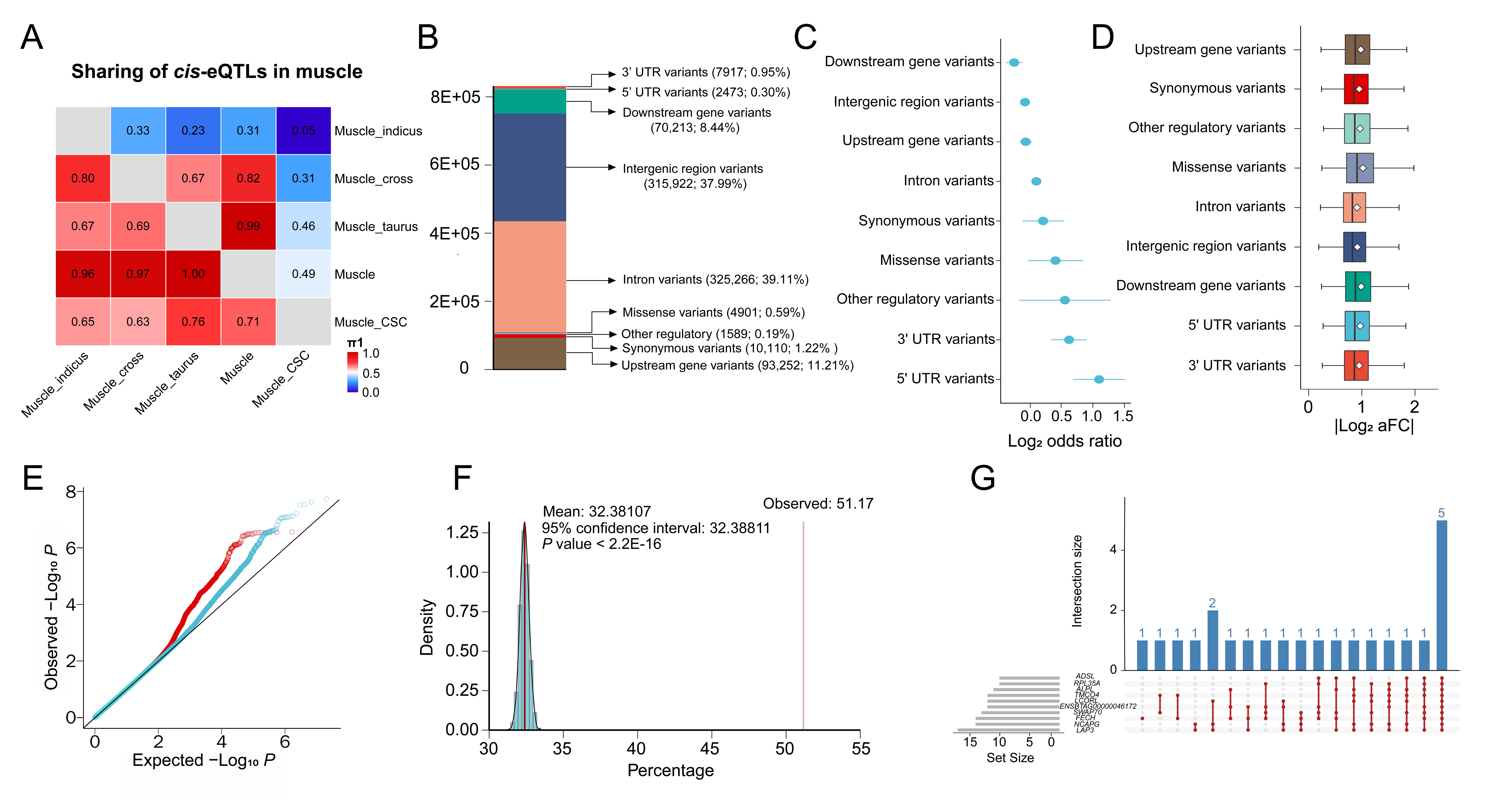

Supplement: qzaf067_Supplementary_Data [file qzaf067_supplementary_data.zip › Figure S5.jpg]
